# Future directions in the treatment of brain metastases: evaluating the role of cellular players in the tumour microenvironment

**DOI:** 10.1007/s00401-026-03011-8

**Published:** 2026-05-19

**Authors:** Chris W. Govaerts, J. Marc C. van Dijk, Anouk van der Hoorn, Frank A. E. Kruyt

**Affiliations:** 1 Department of Medical Oncology, University Medical Center Groningen, University of Groningen, Hanzeplein 1, 9713 GZ Groningen, The Netherlands; 2 Department of Neurosurgery, University Medical Center Groningen, University of Groningen, Groningen, The Netherlands; 3 Department of Radiology, Medical Imaging Center (MIC), University Medical Center Groningen, University of Groningen, Groningen, The Netherlands

**Keywords:** Brain metastasis, Tumour microenvironment, Immune therapy, Cell–cell interaction

## Abstract

Recent clinical trials have demonstrated the efficacy of different modalities of systemic therapy in treating brain metastases, including immune checkpoint inhibitors and various types of targeted therapy. These findings expand treatment options for these intracranial tumours, which are linked to significant morbidity and poor outcomes. Despite these advances, important challenges remain. These include the difficulty of identifying predictors of intracranial response to such therapies. The intracranial activity of anti-tumour agents is dependent on the complex interplay between the cellular components of the tumour microenvironment, consisting of both brain-resident cell types and peripheral immune cells. A deeper understanding of these interactions is essential for identifying mechanisms that contribute to inter- and intratumoural heterogeneity in treatment sensitivity. This review provides a comprehensive overview of the mechanisms by which the constituent cell types of the tumour microenvironment of brain metastases contribute to tumour progression and therapy resistance. Herein, a conceptual model of the relevant biological pathways is outlined along with references to how specific vulnerabilities could be utilised to develop more effective therapeutic strategies.

**Supplementary Information:**

The online version contains supplementary material available at 10.1007/s00401-026-03011-8.

## Introduction

Brain metastasis occurs in up to 30% of cancer patients [12, 25, 62, 119]. It is associated with substantial morbidity and poor survival rates. Symptoms include headache, seizure, focal neurologic deficits and neurocognitive decline [156]. Median overall survival after diagnosis is short at approximately 6–10 months [25, 153]. It is increasingly recognised, however, that variables such as primary tumour type, molecular alterations and lesion number can predict patient-specific prognosis [25, 105, 152, 153]. Notably, the clinical challenge of brain metastasis has grown due to increasing incidence [50, 110]. This is likely due both to treatment and overall survival improvements in cancer generally, and better lesion detection through advances in diagnostic imaging [50, 110].

The central nervous system (CNS) is a unique tumoural location because of its distinctive cellular environment and selectively permeable blood–brain barrier (BBB). This uniqueness also translates to the immune system. The traditional view of the brain as immune privileged has shifted given that there is continuous communication with peripheral immune cells in specialised niches within the CNS [27, 44, 103, 115, 144]. The immune compartment represents a key component of the tumour microenvironment (TME) in brain tumours. The TME describes the milieu of a tumour, and consists of a network of blood vessels, leucocytes and extracellular matrix (ECM) [128]. The TME is particularly complex in brain tumours both because of unique tissue-resident cells including microglia, oligodendrocytes, astrocytes and neurons, as well as the relatively stringent separation of the CNS from the periphery.

The assumption that many systemic agents exhibit poor BBB-penetrance is challenged by evidence that metastatic growth can disrupt the functionality of the BBB [164, 187]. This permits the enhanced entry of otherwise more restricted cells and molecules into the parenchymal space [164, 187]. Clinically, this is reflected in the favourable intracranial response rates of tyrosine kinase inhibitors in non-small cell lung cancer (NSCLC) brain metastases [140, 182]. There are similarly encouraging results with immune checkpoint inhibitors (ICI). Multiple clinical trials have demonstrated the intracranial efficacy of nivolumab, pembrolizumab and ipilimumab in melanoma, nivolumab and ipilimumab in renal cell carcinoma (RCC), and nivolumab, ipilimumab and pembrolizumab in NSCLC [40, 43, 56, 85, 120, 161, 165].

Given that systemic therapies for brain metastases are evolving, understanding the TME and its influence on treatment response has become essential. In particular, tumour immunogenicity and sensitivity to ICI-treatment are regulated by interactions between cells within the TME [107]. The nature of these cell-derived determinants of treatment response in brain metastases is an active area of research. Advancing understanding in this area may help clarify why intracranial response rates to systemic agents vary by primary tumour type and why efficacy is often limited to only a subset of patients within a given tumour type. To address these issues, this review examines how the cellular components of the TME contribute to tumour progression and therapy resistance in brain metastases. In this, it expands on previous reviews [22, 24, 47, 127, 139, 155, 158, 163]. Not discussed in detail are the endothelium or BBB, as these topics have been the focus of two recent comprehensive reviews [11, 72]. The cell types covered here include both brain-resident and peripheral populations. Where relevant, outlined is how specific features of these cellular components have been or could be exploited in experimental and approved therapeutic approaches.

## Brain-resident cells

### Neurons

**Fig. 1:**
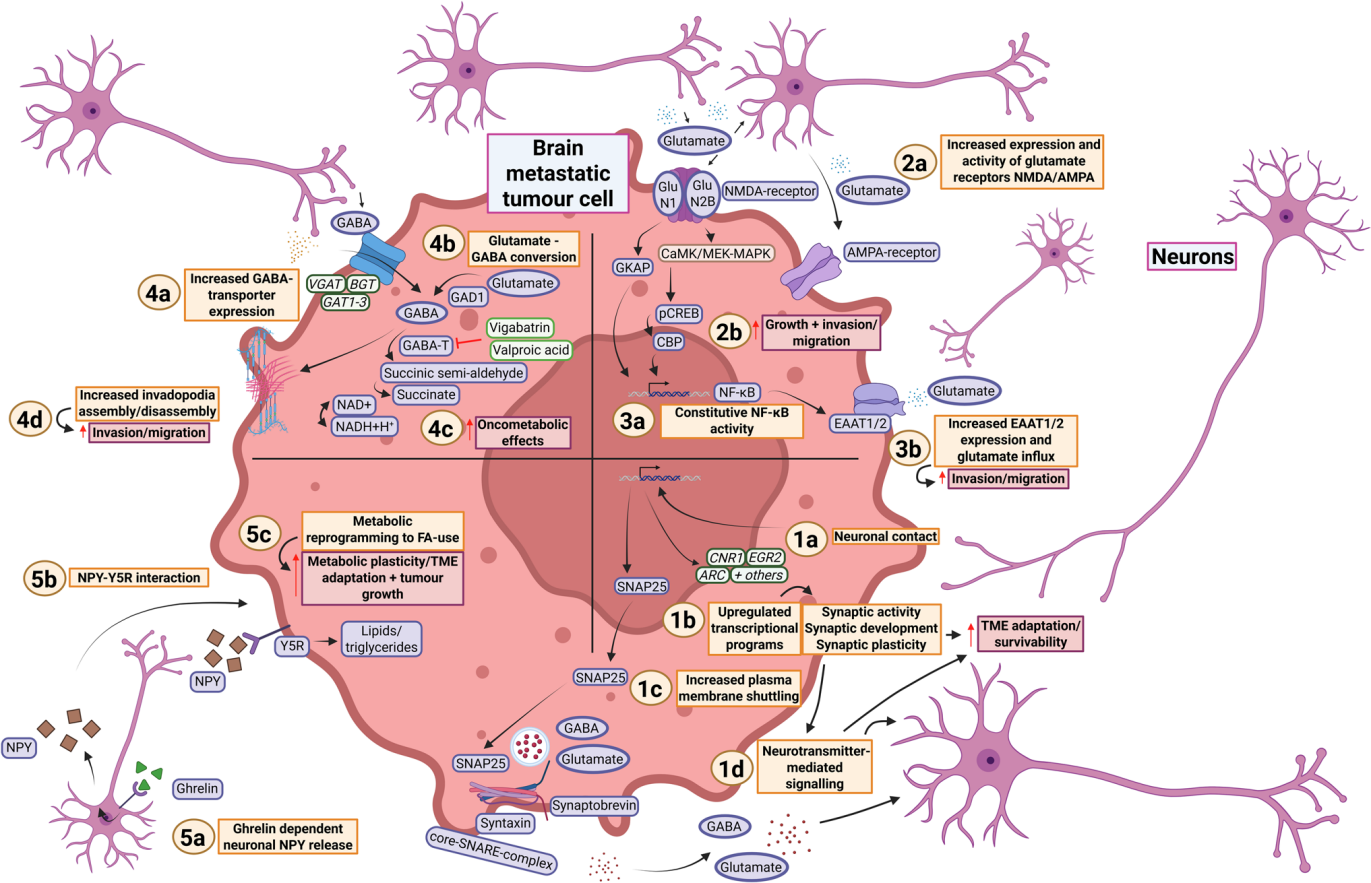
Mechanisms of interaction between tumour cells and neurons. **1a** Neuronal contact leads to the transcriptional re-education of metastatic tumour cells through various mechanisms. **1b** Upregulated transcriptional programmes involve genes linked to synaptic activity, development and plasticity, including *CNR1*, *EGR2* and *ARC*. This reprogramming likely enhances tumour cell adaptation and survivability in the neuronal TME. **1c** Neuronal exposure also increases plasma membrane shuttling of SNAP25, a core component of the SNARE-complex. Other important constituent proteins of the SNARE-complex include synaptobrevin and syntaxin. Increased plasma membrane shuttling of SNAP25 could lead to increased fusion of neurotransmitter containing vesicles and exocytosis promotion. **1d** The transcriptional re-education and increased SNAP25 shuttling likely enhance tumour–neuron communication via neurotransmitter signalling. **2a** Increased expression of the NMDA-receptor (NMDAR) component GluN2B and the AMPA-receptor (AMPAR) allows tumour cells to adapt to the glutamate-rich brain microenvironment. Tumour cells also localise at pseudo-tripartite synapses with neurons, facilitating neuronal communication via NMDAR signalling. **2b** NMDAR signalling promotes tumour cell growth and invasion in various ways, including via the CaMK/MEK-MAPK and GKAP-mediated pathways. Downstream effectors of the CaMK/MEK-MAPK pathway include pCREB and CBP. *Note: these pathways were established downstream of NMDAR signalling in models of extracranial pancreatic neuroendocrine cancer. **3a-3b** Constitutive NF-κB signalling upregulates EAAT1/2 expression, increasing glutamate influx and thereby promoting tumour cell migration and invasion. **4a** Brain metastatic tumour cells show increased mRNA expression of multiple GABA transporters, including *VGAT*, *BGT* and *GAT1-3*, enhancing uptake of GABA from the TME and increasing GABA utilisation independent of GABA-receptor signalling. **4b** Upregulation of GAD1 increases autocrine conversion of glutamate into GABA. **4c** Processing of GABA through the GABA shunt, catalysed through the enzyme GABA-T, leads to oncometabolite production, further promoting progression of the tumour. Vigabatrin and valproic acid inhibit GABA-T, preventing GABA flux into the citric acid cycle and reducing GABA-driven energy production. **4d** GABA is also implicated in invadopodia assembly, enhancing ECM degradation to facilitate tumour cell migration and invasiveness. **5a** Ghrelin is released during fasting and crosses the BBB to bind to neurons, triggering NPY release. **5b-5c** NPY interacts with Y5R on tumour cells, leading to metabolic reprogramming and enhanced fatty acid oxidation. This is thought to increase tumour adaptability within the brain TME. Figure created with BioRender (Toronto, Ontario, Canada). Govaerts, C. (2026) https://BioRender.com/z01r496 is licensed under CC BY 4.0. Colour coding—brown/beige: numbered headings; purple/blue: proteins or protein complexes; brown/brown: pathways; dark green: genes indicating mRNA expression; orange: descriptions of mechanisms; crimson: descriptions of pro-tumour effects; pink: cell types; light green: therapeutic agents. *CNR1* cannabinoid receptor 1, *EGR2* early growth response 2, *ARC* activity regulated cytoskeleton-association protein, *SNAP25* synaptosomal-associated protein 25, *SNARE* soluble N-ethylmaleimide-sensitive factor attachment protein receptor, *GABA* gamma-aminobutyric acid, *TME* tumour microenvironment, *NMDA* N-methyl-D-aspartate receptor, *AMPAR* α-amino-3-hydroxy-5-methyl-4-isoxazolepropionic acid receptor, *GluN1* glutamate [NMDA] receptor subunit zeta-1, *GluN2B* glutamate [NMDA] receptor subunit epsilon-2, *CaMK* Ca^2+^/calmodulin-dependent protein kinase, *MEK* mitogen-activated protein kinase (MAPK)/ extracellular-signal regulated kinase (ERK) kinase, MAPK mitogen-activated protein kinase, *(p)CREB* (phosphorylated) cyclic adenosine monophosphate (cAMP) response element-binding protein, *CBP* CREB-binding protein, *GKAP* guanylate kinase-associated protein, *NF-κB* nuclear factor kappa light chain enhancer of activated B cells, *EAAT1/2* excitatory amino acid transporter 1/2, *VGAT* vesicular GABA transporter, *BGT* GABA-betaine transporter, *GAT1-3* GABA transporters 1–3, *GAD1* glutamate decarboxylase 1, *GABA-T* GABA-transaminase, *NAD(H)* nicotinamide adenine dinucleotide (hydrogen), *NPY* neuropeptide Y, *Y5R* neuropeptide Y receptor Y5, *FA* fatty acid, *ECM* extracellular matrix. [16, 39, 41, 74, 89, 90, 106, 111, 114, 137, 159, 166, 180, 183, 189]

Neurons are increasingly recognised as active contributors to the TME in brain metastases, adopting diverse functions that influence tumour behaviour and malignancy (Fig. 1). Advances in the field of cancer neuroscience have enhanced understanding of neuronal interactions, elucidating the cellular and molecular mechanisms underlying the tumour-promoting ‘neural niche’ [101].

#### Neuronal-like phenotypes allow tumour cells to adapt to the brain microenvironment

Adaptation to the brain microenvironment is crucial for metastatic cell survival. Adaptation involves changes at the epigenetic, transcriptomic and proteomic levels to induce ‘neuronal-like’ phenotypes. The following section discusses examples of these phenotypes, which both mediate brain colonisation and facilitate neuronal communication to promote metastatic outgrowth.

In a study by Deshpande and colleagues, early tumour–neuron interactions were modelled in patient-derived brain metastatic breast and lung cancer cells [39]. Neuronal coculture induced tumour cell expression of modulators of synaptic activity and plasticity [39]. These included cannabinoid receptor 1 (*CNR1*), early growth response 2 (*EGR2*) and activity-regulated cytoskeleton-associated protein (*ARC*) (Fig. 1; 1a-b) [39]. Notably this was tumour type specific, as this effect was limited to breast cancer cells [39]. Furthermore, increased membrane shuttling of synaptosomal-associated protein 25 (SNAP25) was found in both tumour cell types (Fig. 1; 1c-d) [39]. SNAP25 is a component of the soluble *N*-ethylmaleimide-sensitive factor attachment protein receptor (SNARE)-complex and mediates neurotransmitter trafficking [74]. Also shown was that breast cancer cells rely for metabolism primarily on paracrine ƴ-aminobutyric acid (GABA) signalling from nearby neurons rather than endogenous GABA [39]. Consistent with this, treatment with valproic acid, a known GABA-targeting anticonvulsant, was effective in brain metastasis xenografts [39].

Neuronal-like phenotypic transition in breast brain metastatic cells has also been demonstrated through overexpression of the neuronal marker nestin (*NES*) [76]. *NES* expression also correlates with brain metastasis incidence in breast cancer patients, suggesting that it confers a survival advantage to tumour cells in the brain [18, 104]. Similar neuronal gene upregulation has been observed in melanoma and NSCLC brain metastatic cells [106, 114]. Single-cell and single-nucleus RNA-sequencing (sc/snRNA-seq) in patient samples have further validated these observations by identifying brain metastasis cell subsets characterised by neural-like gene programmes [16, 159, 183]. Notably, several of these programmes include marker genes similar to those that characterise transcriptional glioma cell subtypes, suggesting convergent survival mechanisms [53, 159].

In summary, recent studies demonstrate that brain metastatic cells of all clinically important primary tumours can acquire a neuronal phenotype. This enhances their adaptation to the brain microenvironment by increasing their responsiveness to neuronal signalling molecules such as GABA, thereby increasing survivability. An outstanding question is whether such phenotypes can emerge prior to brain entry and colonisation, which could represent a treatment target for metastasis prevention. Indeed this has been suggested by the aforementioned single-cell profiling work, which identified shared dedifferentiated neuronal-like cell states in primary and brain metastatic NSCLC [159].

#### Functional consequences of neuronal gene expression in tumour cells

Genes that may drive a neuronal-like phenotype in tumour cells have been investigated at the mechanistic level by several studies, with such insights offering potential novel treatment targets. Examples of this include brain acid soluble protein-1 (BASP1), a neuronal growth-associated protein found at increased levels in NSCLC brain metastases [94]. BASP1 may promote tumour cell proliferation and brain micrometastatic initiation by signalling through epidermal growth factor receptor (EGFR) [94]. Disrupting this signalling loop could offer therapeutic benefit. Another example involves tubulin beta 3 class III (TUBB3), for which gene expression is upregulated in breast brain metastatic cells [76]. TUBB3 is a core component of neuron-specific microtubules and plays an essential role in axon guidance and growth [124]. Knockdown of *TUBB3* reduces tumour cell migration and ECM adhesion in vitro, while also diminishing brain micrometastatic formation in mice [76]. Interestingly, human epidermal growth factor receptor 2 (HER2) was found to regulate *TUBB3* expression, as demonstrated in vitro and supported by correlative analyses in breast cancer patient samples [75, 117]. Bromodomain and extra-terminal domain (BET) inhibition increases *TUBB3* expression by reducing levels of the transcription factor myeloid zinc finger-1 (MZF-1) [75]. This upregulation in turn sensitises metastatic cells to the microtubule targeting drug vinorelbine, supporting the feasibility of TUBB3 as a therapeutic target in brain metastases from HER2+ breast cancer [75].

#### Neurotransmitters and other signalling molecules as mechanisms of tumour–neuron interaction

Neurotransmitters are increasingly recognised as key players in tumour biology. This is well established in primary glial tumours, where they through synaptic signalling contribute to tumour growth [172, 173]. Their involvement has also been demonstrated in several non-CNS neoplasms [109, 154, 160]. In brain metastases, recent studies have advanced mechanistic insight into tumour cell use of neurotransmitters and other neural signalling molecules to enhance survival and proliferation. The evidence is most developed for glutamate and GABA, the principal excitatory and inhibitory neurotransmitters, respectively. These are discussed first, followed by a brief consideration of other signalling molecules.

**Fig. 2:**
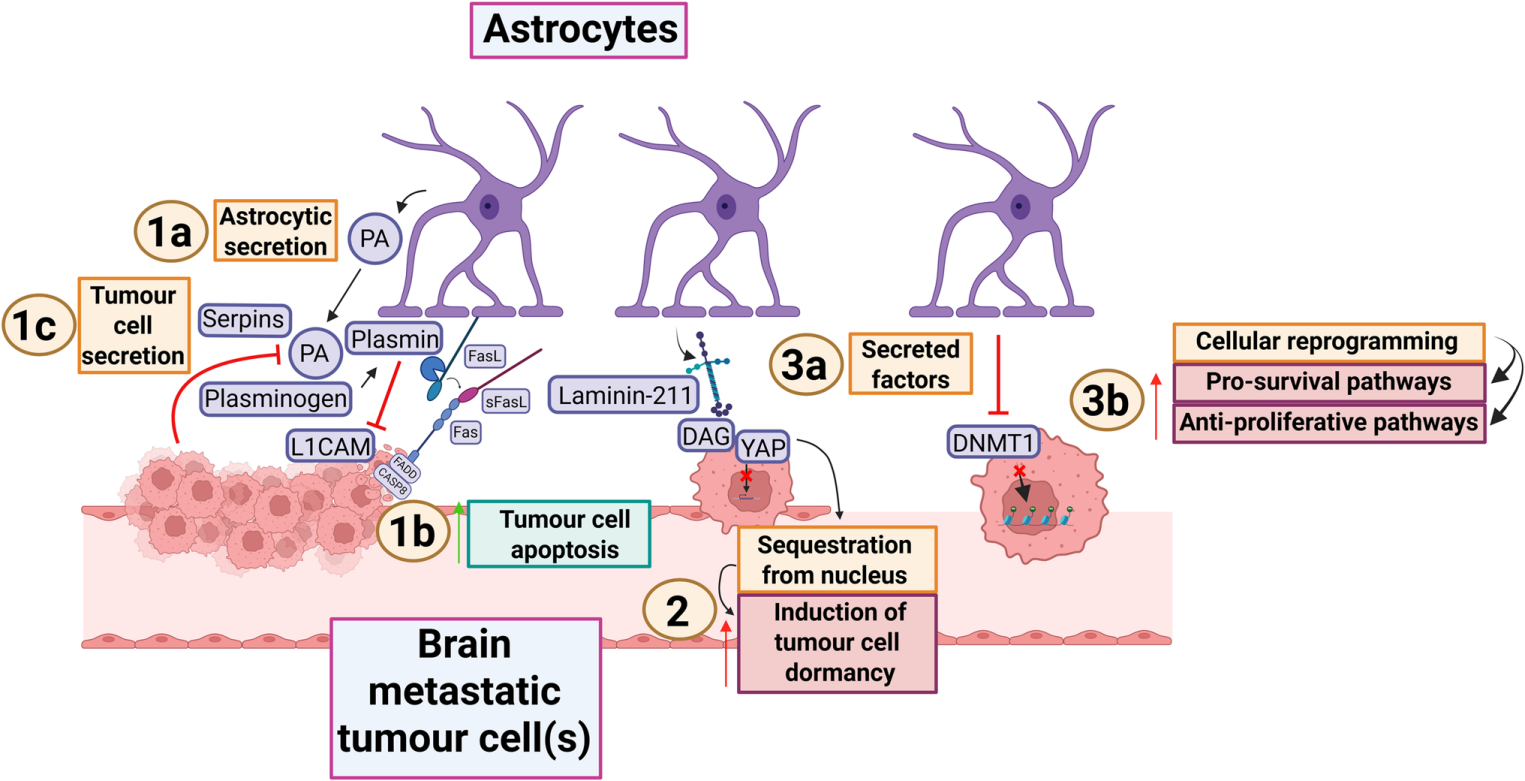
Brain infiltrating tumour cells trigger early astrocytic interactions that affect metastatic growth. **1a** Astrocytes secrete PA into the TME during early contact with invading tumour cells, catalysing the conversion from plasminogen to plasmin. **1b** Plasmin has various anti-tumour effects, including the cleavage of L1CAM on tumour cells, thereby preventing vascular co-option. Further, it engages with FasL on astrocytes, releasing it as sFasL. This in turn promotes apoptosis in tumour cells through Fas-FADD-CASP8 signalling. **1c** Tumour cells can secrete PA inhibitory serpins to mitigate this anti-tumour effect. **2** Soon after cell extravasation, astrocytes interact with tumour cells through laminin-211, which engages with the glycoprotein DAG. This causes sequestration of YAP from the tumour cell nucleus to induce cellular dormancy, thereby promoting pro-survival and anti-proliferative signalling. Tumour cells can remain dormant until permissive conditions allow for metastatic outgrowth. **3a** Similarly, astrocytes can promote cellular dormancy by secreting factors that downregulate DNMT1 in tumour cells. **3b** Suppressed DNMT1 alters tumoural methylation patterns and causes a switch in cellular programming that enhances pro-survival and anti-proliferative pathways. Figure created with BioRender (Toronto, Ontario, Canada). Govaerts, C. (2026) https://BioRender.com/x07w325 is licensed under CC BY 4.0. Note: the PA/serpin pathway has also been illustrated in a figure of a previous review [158]. Colour coding—brown/beige: numbered headings; purple/blue: proteins or protein complexes; orange: descriptions of mechanisms; crimson: descriptions of pro-tumour effects; cyan: descriptions of anti-tumour effects; pink: cell types. *PA* plasminogen activator, *L1CAM* L1 cell-adhesion molecule, *FasL* fas ligand, *sFasL* soluble-FasL, *FADD* fas-associated death domain protein, *CASP8* caspase-8, *DAG* dystroglycan, *YAP* yes-associated protein 1, *DNMT1* DNA methyltransferase 1, *TME* tumour microenvironment [35, 68, 158, 167]

Exposure to the brain microenvironment induces upregulation of glutamate receptors and transporters in multiple tumour types. Glutamate is then used both as a receptor signalling molecule as well as intracellularly for proliferation, migration and invasiveness. This occurs in melanoma via the α-amino-3-hydroxy-5-methyl-4-isoxazolepropionic acid receptor (AMPAR) and in breast cancer through excitatory amino acid transporters 1 and 2 (EAAT1/2) (Fig. 1; 2a; 3a-b) [41, 114]. In breast cancer, this effect is enhanced by constitutive activation of nuclear factor kappa light chain enhancer of activated B cells (NF-κB) (Fig. 1; 3a) [41]. Zeng and colleagues showed that basal-like and triple-negative breast tumours have elevated expression of genes encoding for the *N*-methyl-D-aspartate receptor (NMDAR), which binds to glutamate and glycine ligands (Fig. 1; 2a) [189]. NMDAR signalling is thought to promote cancer cell invasiveness through pathways involving guanylate kinase-associated protein (GKAP) and Ca^2+^/calmodulin-dependent protein kinase (CaMK), mechanisms extensively explored in murine models of pancreatic neuroendocrine tumours (Fig. 1; 2b) [89, 90].

The NMDAR comprises two glycine-binding GluN1 subunits and two glutamate-binding GluN2A–D subunits [88]. Phosphorylated GluN2B is increased in breast brain metastatic samples compared to primary breast tumours [189]. Similar to GABA [39], GluN2B-NMDAR activity in tumour cells is primarily induced through contact with glutamatergic neurons, in this case occurring at pseudo-tripartite synapses [189]. Here, tumour cells align ‘perpendicularly’ with a pre- and post-synaptic neuron pairing, analogous to how perisynaptic astrocytes internally transport glutamate to prevent its extracellular accumulation [186, 189]. Interestingly, Zeng and colleagues showed that GluN2B-knockdown did not impair the passage of tumour cells across the BBB but did reduce metastatic outgrowth [189]. This indicates that NMDAR signalling supports tumour cell survival and growth only after brain entry, further implying that anti-NMDAR therapy would be effective primarily in established lesions. Collectively these findings indicate that brain metastatic tumour cells have an enhanced ability to exploit brain-derived glutamate to promote growth, proliferation and invasion. A similar dependency is observed for GABA, as discussed next.

In breast cancer brain metastasis cells, exogenous GABA has a proliferative effect that is primarily driven by GABA transaminase (GABA-T) through the GABA shunt [111]. The GABA shunt facilitates intracellular GABA recycling for energy production [111]. This is mediated by enhanced GABA influx, as indicated by elevated mRNA expression of vesicular GABA transporter (*VGAT*), GABA transporters 1–3 (*GAT1*–*3*) and the GABA-betaine transporter (*BGT*) (Fig. 1; 4a) [111]. Both melanoma and breast cancer brain metastatic cells also exhibit increased glutamate decarboxylase 1 (*GAD1*) expression, an enzyme that converts glutamate to GABA for metabolic use (Fig. 1; 4b-c) [137]. This upregulation is driven by glial-cell-mediated inhibition of DNA methyltransferase 1 (DNMT1), resulting in reduced methylation of the *GAD1* promoter [137].

Although the precise contribution of the GABA receptors in brain metastasis requires further investigation, several GABA-receptor subunits are upregulated in breast brain metastatic tumour cells [111]. Furthermore, primary breast tumours from patients that develop brain metastases show higher mRNA levels of the pi subunit of the GABA_A_ receptor than primary tumours from patients without brain metastases [147]. In addition, GABA has been implicated in regulating invadopodia assembly and disassembly in brain metastases, suggesting a role in promoting tumour invasion [180]. Williams and colleagues showed that adding GABA in a chick chorioallantoic membrane model with breast brain metastatic cells stimulates invadopodia formation and enhances metastatic seeding (Fig. 1; 4d) [180]. Intravital imaging further showed colocalisation of GABA receptors with invadopodia, suggesting a chemosensory link with GABA [180]. Together, these findings indicate that, similar to glutamate, GABA is also an important driver of metastatic outgrowth in the brain. Beyond these classical neurotransmitters, other neural signalling molecules have also been found to influence brain metastatic behaviour.

 Tyagi and colleagues showed that ghrelin-dependent neuronal secretion of neuropeptide-Y (NPY) upregulates NSCLC brain metastasis cell growth and proliferation by stimulating a metabolic switch from glycolysis to fatty acid oxidation (Fig. 1; 5a-b-c) [166]. This follows the clinical observation that low body-mass-index (BMI) NSCLC patients appear more susceptible to brain metastasis development than normal or high BMI patients [166]. Specifically, circulating plasma ghrelin stimulates brain metastasis growth via activation of its receptor NPY receptor Y5 (Y5R) [166]. Ghrelin, released secondary to caloric restriction, can cross the BBB and stimulate neuronal NPY release to trigger a Y5R signalling cascade in tumour cells (Fig. 1; 5a-b-c) [166]. From this, plasma ghrelin levels may serve as a biomarker for brain metastasis risk profiling in underweight NSCLC patients [166].

#### Reciprocal neuron–tumour cell crosstalk

To date, research has mostly focussed on neuronal effects on cancer via neurotransmitters and other signalling molecules, whereas characterisation of reciprocal interaction is largely lacking. Recently, the effects of brain metastasis on neuronal physiology were studied, providing proof of bi-directional crosstalk [133]. Sanchez-Aguilera and colleagues used machine learning tools to analyse local field potential readings obtained in vivo, demonstrating that brain metastases impact local neural circuitry in distinct ways depending on the histology of the primary tumour [133]. The transcription factor early growth response protein 1 (EGR1) was identified as a possible mediator of these effects through its role in angiogenesis and synaptic plasticity [133]. This implies that the molecular characteristics, and not just the mass effect of tumours, play a crucial role in influencing neuronal activity [133]. This opens the possibility of using non-invasive electrophysiological approaches to provide diagnostic information about the underlying tumour on an individual-patient basis [133].

#### Therapeutic insights

As noted above, brain metastases exploit GABA and glutamate in neuronal niches for progression. This dependency has therapeutic relevance, illustrated by brain metastatic cells being sensitive to the anticonvulsants vigabatrin and valproic acid [39, 111, 137]. Both drugs inhibit GABA-T directly or indirectly, a key downstream effector of GAD1 and a rate limiter for GABA flux via the GABA shunt into the citric acid cycle (Fig. 1; 4b-c) [10, 200]. Anticonvulsants are not the only CNS-targeting drugs that can potentially be repurposed. Recent reports provide preclinical evidence that antipsychotics exert anti-brain metastasis effects. Trifluoperazine, fluphenazine, clozapine and sertindole each to differing extents inhibit proliferation and viability of melanoma and breast brain metastatic cells in vitro and in vivo [179, 184, 194, 196]. Further studies are needed to validate these observations and elucidate the underlying mechanisms of cytotoxicity. It also remains unclear whether the anti-tumour effects of these agents are more pronounced because of the previously described adoption of neuronal-like phenotypes of brain metastatic cells or whether they simply reflect their inherent ability to suppress cellular metabolism. Moreover, the clinical implications for brain metastasis patients remain unclear.

In summary, recent research has shown that tumour cells undergo phenotypic changes that allow their survival in a neuron-rich environment. This involves the adoption of ‘neuronal-like’ characteristics through epigenetic and transcriptional modification. The presented evidence indicates that this occurs under the influence of the neuronal TME, resulting in the upregulation of processes such as neurotransmitter trafficking and metabolism. This predisposes tumour cells to exploit mechanisms such as glutamate-NMDAR signalling in pseudo-tripartite synapse conformations or take advantage of GABA-rich environments for metabolism, growth and migration. It is notable that most of the neurotransmitter-mediated effects have been observed in breast cancer, although it is not clear to what degree this reflects tumour-type specificity or reduced investigative effort into for example melanoma or NSCLC. Future research should investigate this gap while also uncovering the extent to which neuronal adaptation occurs before brain colonisation, the tumour- and microenvironment-specific factors that drive it, and the therapeutic potential of targeting relevant pathways.

### Astrocytes

Astrocytes are more numerous than neurons in the CNS and have diverse functions [1, 79, 150, 168]. A common hallmark of CNS-pathology is reactive astrogliosis, in which astrocytes undergo morphological and functional change to become ‘reactive astrocytes’ [45, 142]. Reactive astrocytes associate early with invading cancer cells and this interaction persists throughout the development of metastatic lesions [63, 97, 142]. This section outlines the diverse roles of astrocytes in brain metastasis, beginning with the induction of tumour cell dormancy. This is followed by a discussion of the role of astrocytes in immune evasion and the various mechanisms of direct astrocyte–tumour cell communication leading to both pro- and anti-tumour effects.

#### Early astrocyte–tumour cell interactions and dormancy induction

Astrocytes may initially counteract tumour spread to the brain. They secrete plasminogen activator (PA) to kill metastatic tumour cells via plasmin shortly after BBB penetration, which lung and breast tumour cells can resist through PA inhibitory serpin expression (Fig. 2; 1a-b-c) [167]. Whether this function is limited to a subset of astrocytes, or whether this represents an early phase before astrocytes transit to a pro-tumour state during tumour evolution is not well understood.

One of the proposed mechanisms for brain metastasis development is that tumour cell invasion occurs early in the systemic disease process but that once disseminated, cells remain dormant [4, 99, 135]. This dormancy phase is considered a rate-limiting step in metastatic development, with clinically detectable lesions forming only under permissive conditions [4, 99, 135]. In breast cancer brain metastasis models, astrocytes were found to induce tumour cell dormancy by embedding insoluble ECM glycoproteins such as laminin-211 into the brain parenchymal basement membrane (Fig. 2; 2) [35]. Tumour cell quiescence is maintained after metastatic seeding through the interaction between laminin-211 and the non-integrin cell-adhesion receptor dystroglycan [35]. This interaction sequesters the transcription factor yes-associated protein-1 (YAP) from the nucleus, leading to the suppression of tumour outgrowth (Fig. 2; 2) [35]. The importance of YAP-signalling for escaping dormancy is supported by frequent *YAP1* gene amplifications in established lung adenocarcinoma brain metastases [143]. A parallel dormancy mechanism proposed by Hirata and colleagues involves the inhibition of DNMT1 (Fig. 2; 3a-b) [68]. Astrocyte-conditioned media could reduce DNMT1 levels in multiple tumour types, resulting in cell cycle suppression and causing tumour cells to remain indolent as single cells [68]. This was attributed to demethylation of DNMT1 target genes such as L1 cell-adhesion molecule (*L1CAM*) [68].

These insights suggest that once tumour cells escape dormancy, they can establish persisting lesions. The spatial orientation of microenvironmental cells relative to tumour cells within these lesions is known to influence tumour progression, heterogeneity and treatment response [70, 174]. Recent studies found that in contrast to glioma cells, which are more infiltrative and engage with astrocytes throughout the tumour, interaction with brain metastatic cells is predominantly limited to the tumour periphery [36, 77]. This pattern appears to be in part dependent on tumour type and on the invasion and migratory capacity of tumour cells [36, 52]. For example, early-stage HER2+ breast cancers restrict astrocyte infiltration to the periphery, whereas triple-negative tumours enable deeper infiltration [52].

Taken together, in multiple tumour types astrocytes can induce tumour cell dormancy after brain entry to induce their survival. This is used as a long-term repressive mechanism prior to definitive colonisation and further progression of the metastatic lesion, whose ongoing interaction with astrocytes is influenced by tumour type and architecture. Recent work has made great progress in characterising the immunomodulatory nature of this interaction in established metastatic lesions, which is discussed in the next section.

#### Astrocytes and immune evasion

**Fig. 3:**
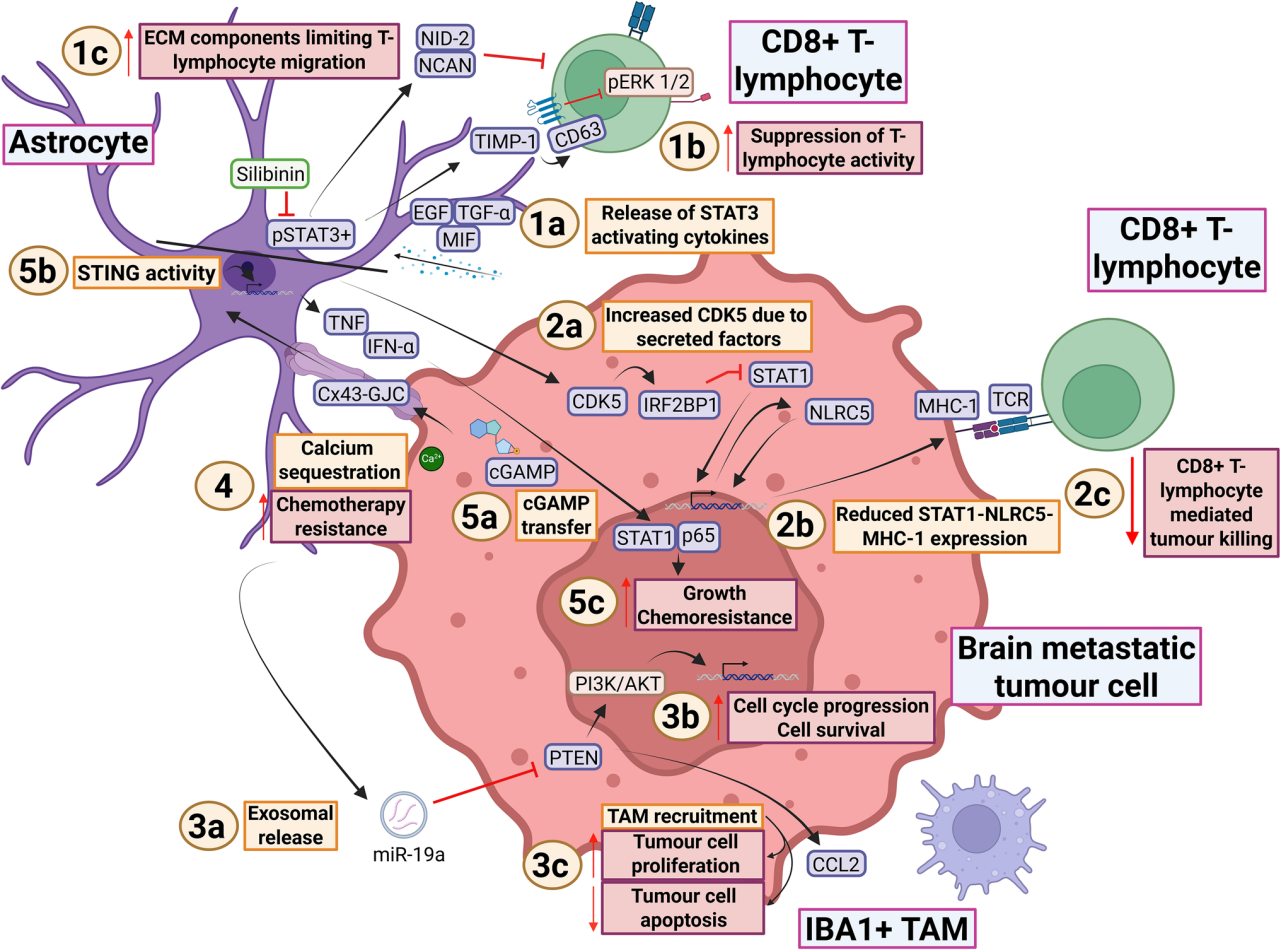
Metastasis promoting interactions between astrocytes and tumour cells. **1a** Co-localisation of pSTAT3+ astrocytes to tumour cells is a notable feature of brain metastasis, which occurs through the secretion of STAT3-activating cytokines such as MIF, TGF-α and EGF. **1b** pSTAT3+ astrocytes contribute to tumour cell survival through the suppression of T-lymphocyte activity, occurring directly through immunosuppressive factors such as TIMP-1, which binds to CD63 on T-lymphocytes to impact their effector functions through pERK 1/2 modulation. **1c** Astrocytes also reduce T-lymphocyte activity by secreting ECM components such as NID-2 and NCAN, which form a physical barrier to T-lymphocyte chemotaxis and reduce infiltration and anti-tumour functionality. Silibinin, a STAT3 inhibitor, is under investigation as a potential therapeutic agent in brain metastases. **2a** Interaction between astrocytes and tumour cells enhances CDK5 signalling. **2b** CDK5 activity reduces MHC-1 expression through the IRF2BP1-STAT1-NLRC5 pathway, where STAT1 is suppressed by IRF2BP1. This reduces NLRC5-stimulated MHC-1 expression, thereby diminishing tumour cell antigen presentation. **2c** Reduced MHC-1 expression inhibits anti-tumour cytotoxic T-lymphocyte function. **3a** In established lesions, astrocytes secrete exosomes containing microRNA-19a. After being taken up by tumour cells, the 3’ untranslated region of *PTEN* is targeted to cause its suppression. **3b** Although not definitively demonstrated in [190], PTEN loss should increase PI3K/AKT signalling, promoting cell cycle progression and tumour cell survival. **3c** PTEN inhibition also enhances CCL2 secretion, promoting recruitment of pro-tumour macrophages that enhance tumour cell proliferation and suppress apoptosis, respectively. **4** Astrocytes form Cx43 gap junctions with tumour cells, enabling calcium sequestration to astrocytes. This has been found to promote chemotherapy resistance in tumour cells by interfering with pro-apoptotic pathways. **5a-b** The transfer of cGAMP from tumour cells to astrocytes via Cx43 gap junctions upregulates genes downstream of STING, causing the release of IFN-α and TNF. **5c** IFN-α and TNF release by astrocytes enhances tumour cell growth and chemotherapy resistance via STAT1 and p65. Figure created with BioRender (Toronto, Ontario, Canada). Govaerts, C. (2026) https://BioRender.com/23y5zj7 is licensed under CC BY 4.0. Note: the pathways involving microRNA-19a/PTEN, Cx43-mediated cGAMP transfer and pSTAT3+ immunomodulation have also been illustrated in figures of previous reviews [47, 127, 158]. The spatial arrangement of this figure was informed in part by Figure 4 in [158]. Colour coding—brown/beige: numbered headings; purple/ blue: proteins or protein complexes; brown/brown: pathways; orange: descriptions of mechanisms; crimson: descriptions of pro-tumour effects; pink: cell types; light green: therapeutic agents. *(p)STAT3* (phosphorylated) signal transducer and activator of transcription 3, *EGF* epidermal growth factor, *TGF-α* transforming growth factor alpha, *MIF* macrophage migration inhibitory factor, *TIMP-1* tissue inhibitor of metalloproteinase (TIMP) metallopeptidase inhibitor 1, *CD63* cluster of differentiation 63, *(p)ERK* (phosphorylated) extracellular-signal regulated kinase, *NID-2*: nidogen-2, *NCAN* neurocan, *ECM* extracellular matrix, *CDK5* cyclin-dependent kinase 5, *IRF2BP1* interferon regulatory factor 2 binding protein 1, *NLRC5* nucleotide-binding oligomerization domain (NOD)-like receptor family caspase recruitment domains (CARD) domain containing 5, *MHC-1* major histocompatability complex 1, *TCR* T-cell receptor, *miR-19a* microRNA-19a, *PTEN* phosphatase and tensin homolog, *PI3K* phosphoinositide 3-kinase, *AKT* protein kinase B, *CCL2* chemokine (C–C motif) ligand 2, *IBA1* ionised calcium-binding adapter molecule 1, *TAM* tumour-associated macrophage, *Cx43* connexin 43, *GJC* gap-junction channel, *cGAMP* cyclic guanosine monophosphate-adenosine monophosphate, *STING* stimulator of interferon genes, *IFN-α* interferon alpha, *TNF* tumour necrosis factor, *p65* transcription factor p65, *TME* tumour microenvironment [28, 36, 47, 95, 116, 122, 123, 127, 158, 188, 190]

Recent reports implicate astrocytes in inducing an immunosuppressive microenvironment in brain metastases. A population of phosphorylated signal transducer and activator of transcription 3 (pSTAT3)-positive reactive astrocytes tends to co-localise to brain metastatic cells and is a critical determinant of immunogenicity in multiple tumour types (Fig. 3; 1a) [36, 116, 122, 123]. This is related to the secretome of these astrocytes, which suppresses T-lymphocyte infiltration through immunosuppressive factors such as vascular endothelial growth factor A (VEGF-A), tissue inhibitor of metalloproteinases-1 (TIMP-1) and lipocalin-2, as well as ECM components that may form a physical barrier to CD8+ T-lymphocyte engagement, such as nidogen-2 (NID-2) and neurocan (NCAN) (Fig. 3; 1b-c) [123]. Notably, brain metastasis cells themselves can trigger STAT3 upregulation in astrocytes with STAT3-activating cytokines [123].

These findings were expanded through interrogation of TIMP-1, found in the secretome of pSTAT3-positive astrocytes [122]. A non-canonical function of TIMP-1 was identified through its binding to CD63 on CD8+ T-lymphocytes (Fig. 3; 1b) [122]. This leads in T-lymphocytes to reduced expression of cytotoxicity-associated genes and attenuated killing of tumour cells [122]. This effect is mediated by downstream ERK 1/2 phosphorylation in T-lymphocytes [122]. This underpins the rationale of combining the pSTAT3 targeting agent silibinin with ICI therapy, which may result in higher efficacy than either treatment alone [122]. This combined treatment offers a potential follow-up to the ongoing phase 2 trial where silibinin monotherapy is being tested in brain metastases of NSCLC and breast cancer post gross-total resection (NCT05689619) [122].

While pSTAT3-positive astrocytes are central to astrocyte-mediated immunosuppression, there are multiple other ways in which astrocytes influence tumour immune status. Not accounting for these could hinder therapeutic strategies aimed at this axis. Examples include astrocyte-induced upregulation of cyclin-dependent kinase 5 (CDK5) in breast cancer cells, downregulating major histocompatibility complex 1 (MHC-1) expression (Fig. 3; 2a-b-c) [188]. In other work, amyloid-beta (Aß) production by melanoma was found to both promote metastasis to the brain and sustain tumour colonisation [81]. Interestingly, Aß could induce local reactive astrogliosis and reduce complement component 3 (C3) expression in astrocytes, thereby preventing pro-inflammatory A1-type polarisation [81]. Astrocyte-derived C3 also recruited phagocytic pro-inflammatory microglia, thereby promoting subsequent anti-tumour effects [81].

In short, astrocytes shape the immune TME through diverse mechanisms, with a pSTAT3-positive subpopulation playing a central role by directly impairing T-lymphocyte effector function in multiple primary tumour types. Combining immune checkpoint therapy and pSTAT3 inhibition could be a feasible therapeutic option in brain metastases, although alternative immunosuppressive mechanisms warrant consideration. There are also other means of communication between astrocytes and tumour cells separate from immunomodulation, which are discussed in the following section.

#### Reciprocal astrocyte–tumour cell communication

The immunoregulatory axis is only one part of a broader network of crosstalk between astrocytes and tumour cells, which results in both pro- and anti-tumour effects. Recent studies have focussed particularly on exosome-mediated signalling and gap-junction communication. Breast brain metastatic cells can undergo reversible loss of phosphatase and tensin homolog (*PTEN*) through astrocyte-derived exosomes containing microRNA-19a (miR-19a) [190]. This leads to *PTEN* reduction and increased tumour outgrowth (Fig. 3; 3a-b) [190]. miR-19a also stimulates chemokine (C–C motif) ligand 2 (CCL2) signalling, promoting infiltration of pro-tumour myeloid cells (Fig. 3; 3c) [190]. Notably, astrocytes can also use exosome microRNA transfer to inhibit oncogenic drivers and reduce tumour outgrowth. This has been demonstrated with miRNA-142-3p, which downregulates the transient receptor potential ankyrin-1 (TRPA1)–fibroblast growth factor receptor 2 (FGFR2) complex in lung adenocarcinoma cells [15].

**Fig. 4:**
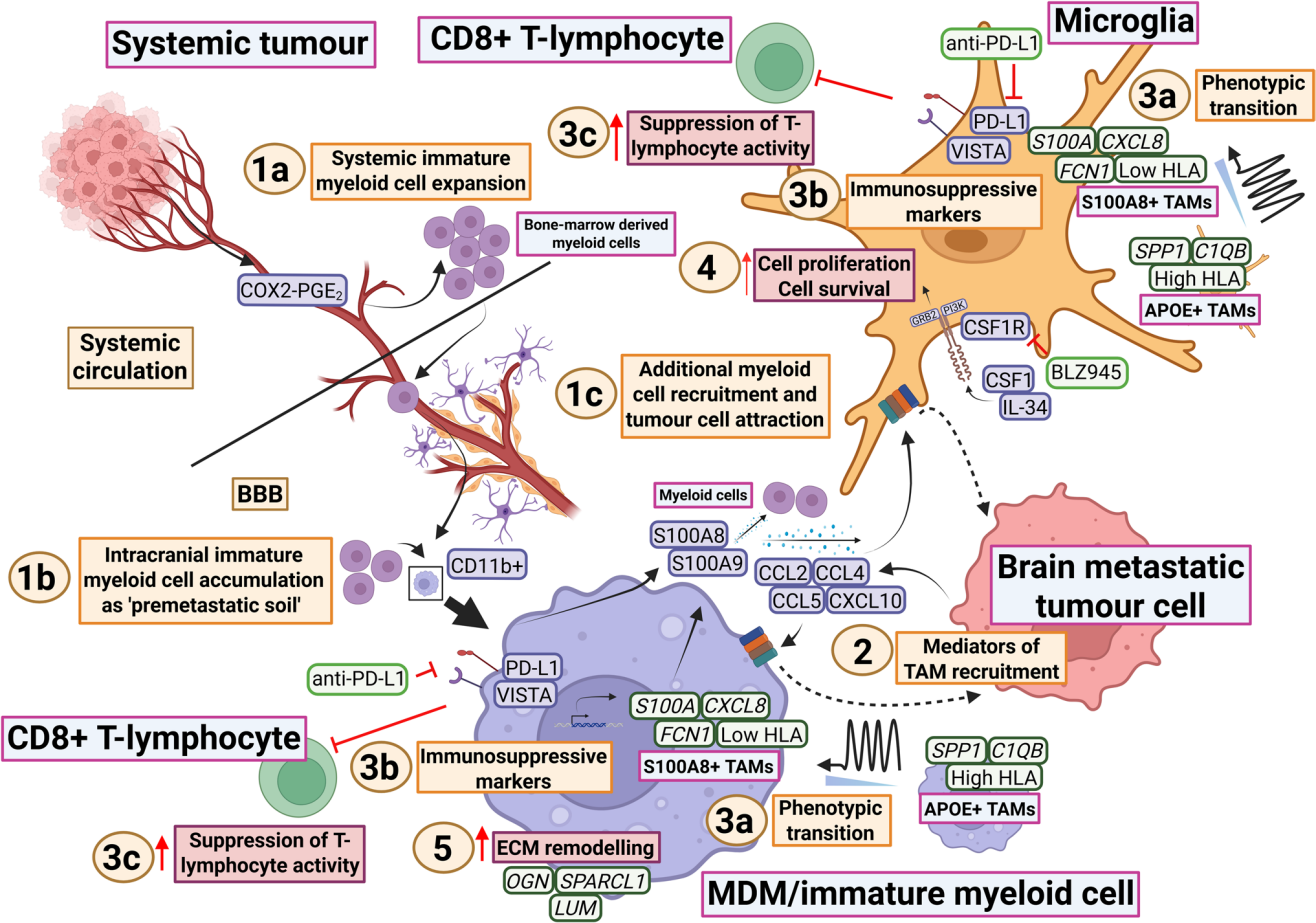
TAM recruitment and activity in the brain metastasis TME. **1a** Systemic extracranial tumours secrete COX2-PGE_2_ to induce expansion of immature bone-marrow-derived myeloid cells. **1b** COX2-PGE_2_ release promotes intracranial accumulation of CD11b+ immature (monocyte and neutrophil-like) bone marrow derived myeloid cells across the BBB, establishing a tumour-permissive ‘premetastatic soil’. **1c** The accumulated CD11b+ cells function to recruit additional myeloid cells and tumour cells through the secretion of S100A8 and S100A9. **2** Tumour cell-derived CCL2, CCL4, CCL5 and CXCL10 mediate TAM recruitment. **3a** scRNA-seq analyses have demonstrated phenotypic diversity of TAMs, which span a spectrum from anti-tumour ‘APOE+ TAMs’ to pro-tumour ‘S100A8+ TAMs’. APOE+ TAMs are characterised by the expression of *SPP1*, *C1QB* and HLA-type genes, whereas S100A8+ TAMs show elevated expression of S100A-family genes, *CXCL8* and *FCN1* and the downregulation of HLA-type genes. **3b** Immunosuppressive TAMs also upregulate surface markers PD-L1 and VISTA to cause local T-lymphocyte suppression. **3c** These various immunomodulating factors contribute to the inhibition of T-lymphocyte activity. **4** In both microglia and MDMs (*only depicted on microglia due to lack of space), CSF1R activation by microenvironmental CSF1 and IL-34 contributes to TAM cell proliferation and survival. CSF1R can be directly inhibited with the agent BLZ945. **5** MDMs contribute to ECM remodelling, as indicated by increased gene expression of *OGN*, *SPARCL1* and *LUM*. This may enhance tumour cell migration and invasion. Figure created with BioRender (Toronto, Ontario, Canada). Govaerts, C. (2026) https://BioRender.com/b3a35v5 is licensed under CC BY 4.0. Note: the markers of immunosuppressive TAMs (PD-L1, VISTA), pathway of CSF1R-mediated TAM survival and the collection of CD11b+ myeloid cells as premetastatic soil have also been illustrated in figures of prior reviews [47, 127, 139, 158]. Colour coding—brown/beige: headings; purple/blue: proteins or protein complexes; dark green: genes indicating mRNA expression; orange: descriptions of mechanisms; crimson: descriptions of pro-tumour effects; pink: cell types; light green: therapeutic agents. *COX2* cyclooxygenase-2, *PGE*_*2*_ prostaglandin E2, *BBB* blood–brain barrier, *CD11b* cluster of differentiation 11b, *S100* S100 calcium-binding protein, *APOE* apolipoprotein E, *TAM* tumour-associated macrophage, *SPP1* osteopontin, *C1QB* complement C1q B chain, *HLA* human leucocyte antigen, *CXCL* chemokine (C–X–C motif) ligand, *FCN1* ficolin 1, *PD-L1* programmed death-ligand 1, *VISTA* V-domain Ig suppressor of T-cell activation, *MDM* monocyte-derived macrophage, *CCL* chemokine (C–C motif) ligand, *CSF1R* colony-stimulating factor 1 receptor, *IL-34* interleukin-34, *ECM* extracellular matrix, *OGN* osteoglycin, *SPARCL1* SPARC like 1, *LUM* lumican, *TME* tumour microenvironment, *scRNA-seq* single-cell RNA sequencing [8, 20, 47, 52, 57, 61, 82, 83, 96, 127, 129, 139, 141, 158]

Examples of gap-junction-mediated communication include the transfer and sequestration of calcium to astrocytes via connexin 43 (Cx43), which confers chemotherapy resistance to brain metastasis cells in melanoma (Fig. 3; 4) [95]. Tumoural protocadherin-7 (PCDH7) can also interact with Cx43 to promote transfer of the second messenger 2′3’-cyclic GMP-AMP (cGAMP) from lung and breast cancer cells to astrocytes (Fig. 3; 5a-b) [28]. This directs astrocytes to upregulate genes downstream of stimulator of interferon genes (STING) and release interferon (IFN)-α and tumour necrosis factor (TNF) [28]. Brain metastasis cell survival is consequently enhanced through STAT1 and p65/NF-κB signalling (Fig. 3; 5c) [28].

In summary, the diversity in the physiological function of astrocytes is recapitulated in the multiformity of their interactions with cancer cells. They display high plasticity, contributing to both pro- and anti-tumour processes involving dormancy induction, immunomodulation, exosomal transfer and gap-junction communication. It would therefore be too simplistic to state that they are predominantly tumour promoting, especially considering that the underlying mechanisms and mediators that drive their plasticity have not been fully elucidated. Future efforts should be directed towards uncovering what the influence is of defined microenvironmental niches on astrocyte behaviour and whether specific tumour types or subclones within a tumour are predictive of pro-tumour polarisation tendencies.

### Microglia and monocyte-derived macrophages

Tumour-associated macrophages (TAMs) in brain tumours include microglia and monocyte-derived macrophages (MDMs). Microglia are derived from myeloid progenitors in the embryonic yolk sac to become resident in the brain, whereas MDMs originate from the bone marrow and enter the brain from the peripheral circulation [19, 54]. Although MDMs are not brain-resident, they will be discussed together with microglia in this section given their partially overlapping characteristics. TAM recruitment and phenotypic heterogeneity, which includes transitions between functional subpopulations, are first reviewed. This is followed by an overview of the mechanistic basis of TAM pro- and anti-tumour function by detailing their roles in immunoregulation and ECM remodelling.

#### TAM recruitment

TAMs have received considerable interest in gliomas due to their abundance in the TME and their role in promoting malignancy and treatment resistance [2, 125]. Similarly, they also comprise a significant portion of the tumour mass in brain metastases [8, 38, 51, 57, 60, 77, 83, 102]. Past work has enhanced understanding of the mechanisms driving the extensive recruitment of TAMs to tumour regions. Primary breast tumours, through cyclooxygenase-2 (COX2) and prostaglandin E_2_ (PGE_2_), induce CD11b+ myeloid cell accumulation (both monocyte-like and neutrophil-like cells) in the brain as ‘premetastatic soil’ to facilitate tumour cell colonisation (Fig. 4; 1a-b) [96]. These myeloid cells then both recruit additional myeloid cells and attract tumour cells through production of S100 calcium-binding (S100) proteins, including S100A8 and S100A9 (Fig. 4; 1c) [96]. Other mediators of TAM recruitment across multiple brain metastatic tumour types include tumour- and myeloid-cell-derived CCL2, CCL4, CCL5 and C–X–C motif chemokine ligand-10 (CXCL10), with the majority of recruited cells consisting of microglia rather than MDMs (Fig. 4; 2) [52, 61, 129, 141].

#### Phenotypic heterogeneity and TAM transition from ‘anti’ to ‘pro-tumour’

S100 proteins therefore appear to have a role in TAM recruitment. They function as intracellular calcium sensors but also influence a variety of extracellular regulatory processes [20]. scRNA-seq studies across multiple brain metastatic tumour types have identified distinct TAM subpopulations characterised by high expression of S100A-linked genes [8, 57]. These TAMs resemble myeloid-derived suppressor cells, with elevated *CXCL8* and reduced human leucocyte antigen (HLA)-related gene expression [8, 57]. They represent one end of a dynamic differentiation continuum of phenotypes with another subpopulation, termed apolipoprotein E (APOE)-positive macrophages, which conversely display much higher HLA- and antigen-presentation activity (Fig. 4; 3a-b-c) [57]. TAMs are thought to transition from an ‘APOE-positive’ to ‘S100A8-positive’ phenotype (Fig. 4; 3a) [57]. This may reflect tumour cell-driven education of TAMs over time from a predominately anti- to pro-tumour state (Fig. 4; 3b-c). These findings also suggest that the functionality and behaviour of TAMs in brain metastases are more complicated than the traditionally postulated ‘M1/M2’ dichotomy and that there is a high degree of phenotypic heterogeneity in these cells [57]. Interestingly, studies have demonstrated the utility of targeting S100A9 in lung cancer to prevent rates of brain metastasis [17]. This reflects the fact that S100 expression is not limited to TAMs but is also upregulated in tumour cells [17]. Whether S100 targeting in tumour cells has an effect on pro-tumour TAM recruitment remains to be examined.

The importance of phenotypic transition in TAMs raises questions about the factors that drive these changes. Separate scRNA-seq findings in a lung cancer brain metastasis model indicate that transcriptional reprogramming of TAMs occurs early under the influence of tumour cells and is sustained throughout tumour progression [141]. Corroborating this, intravital microscopy through cranial windows has shown that TAMs from lung, breast and melanoma models interact quickly with extravasated tumour cells and that these interactions are largely phagocytic, but that TAMs progressively undergo functional changes towards tumour-promoting roles [126, 145, 195].

Although these findings support a model of TAM transition from an anti- to pro-tumour state, there are observations that challenge this paradigm. An example of this is that inhibition of colony-stimulating factor 1 receptor (CSF1R) with BLZ945 prevents early microglia-tumour cell interactions at brain invasion sites and reduces breast tumour growth in mice, suggesting a tumour-supporting role of microglia in early stages of metastatic colonisation (Fig. 4; 4) [82].

An additionally important determinant of TAM phenotype is the spatial context. In a breast brain metastatic cancer model, tumour cells were transfected with a plasmid encoding a secretable fluorescent mCherry protein, which could be endocytosed by neighbouring cells [102]. This showed that a population of CD206+ macrophages that were mCherry positive, suggesting prior contact or ongoing communication with tumour cells, could be found outside the metastatic niche near distant CD31+ vasculature [102]. The precise function of this macrophage subpopulation at some distance from the tumour is not yet clear, although CD206 expression is associated with M2-like polarisation [102]. This observation highlights that a snapshot of spatial proximity at a single time point is likely insufficient to fully explain tumour cell–TME interactions. A deeper understanding of how temporal dynamics relate to changing cellular positions within defined spatial niches is required. Longitudinal monitoring of TME changes may be of clinical use in defining treatment responders from non-responders. PET-based imaging biomarkers represent promising tools for this purpose, as has been summarised in a recent review [37].

In summary, TAM recruitment to the brain metastasis TME is driven by multiple factors, including various chemo/cytokines and S100 proteins. S100 expression defines a subgroup of TAMs with pro-tumour functionality that likely represents the end-point of a broader phenotypic transition from more tumour inhibitory states. Better understanding of transition states and the role of spatial niches is required, however, as illustrated, respectively, by the fact that CSF1R inhibition reduces early tumour growth as well as the changing positional dynamics of CD206+ macrophages. Next, the diverse findings with respect to pro- and anti-tumour TAM functionality in the brain metastasis TME are highlighted.

#### Immunoregulatory role of TAMs

As outlined above, both microglia and MDMs show highly variable polarisation states in brain metastases. They show gene expression signatures reminiscent of both classical M1 (IFN-γ high) and M2 (IL-4 high) phenotypes [83, 108]. Microglia appear more involved in innate immune sensing, with high gene expression of complement cascade components and Toll-like receptor signalling molecules, whereas MDMs engage more in antigen presentation and adaptive immunity [141]. Already discussed extensively is that TAMs transit to a pro-tumour state as a function of time and can adopt immunoinhibitory roles. For example, TAMs can co-express V-domain Ig suppressor of T-cell activation (VISTA) and programmed death-ligand 1 (PD-L1), suggesting a role for these cells in suppressing T-lymphocyte response (Fig. 4; 3b-c) [61]. In melanoma brain metastasis models, theimmunosuppressive functionality of microglia specifically has been linked to high NF-κB levels [129]. This can be exploited therapeutically, as combined inhibition of the NF-κB complex and PD-L1/cytotoxic T-lymphocyte-associated protein 4 (CTLA-4) has demonstrated greater efficacy in reducing metastatic burden than anti-PD-L1/CTLA-blockade alone [129].

#### TAMs engage in ECM remodelling

**Fig. 5:**
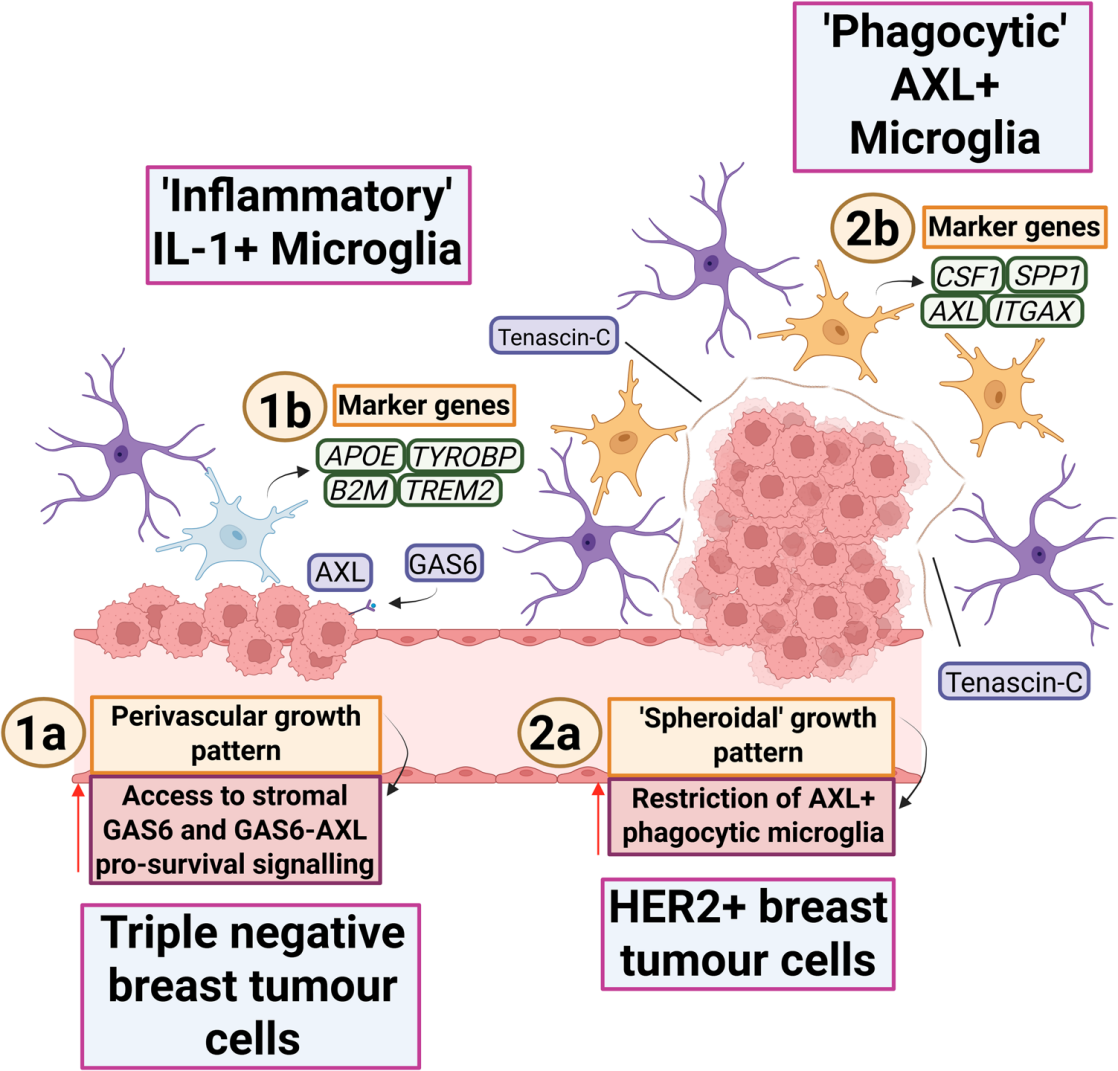
Interactions between tumour cells and microglia are influenced by tumour subtype and architecture, as shown by Gan and colleagues [52]. **1a** Triple-negative breast tumour cells adopt a perivascular pattern of growth and maintain diffuse microglial contact after metastatic seeding. This is thought to increase access to stromal GAS6 and thereby upregulate pro-survival GAS6-AXL signalling at the tumour cell membrane. **1b** Microglia engaged with triple-negative breast cancer cells adopt an ‘inflammatory’ IL-1+ phenotype, characterised by the expression of marker genes such as *APOE*, *TYROBP*, *B2M* and *TREM2*. **2a** HER2+ breast tumour cells grow as spheroids after brain invasion, which is promoted through extensive ECM remodelling and the deposition of ECM proteins, including tenascin-C. Phagocytic AXL+ microglia are restricted from the growing tumour mass. **2b** Microglia are re-educated towards a ‘phagocytic’ AXL+ phenotype due to tenascin-C-TLR4 signalling on the microglial cell membrane. This phenotype is characterised by the elevated expression of marker genes such as *CSF1*, *SPP1*, *AXL* and *ITGAX*. Figure created with BioRender (Toronto, Ontario, Canada). Govaerts, C. (2026) https://BioRender.com/pxytj8z is licensed under CC BY 4.0. Colour coding—purple/blue: proteins or protein complexes; dark green: genes indicating mRNA expression; orange: descriptions of mechanisms; crimson: descriptions of pro-tumour effects; pink: cell types. *GAS6* growth arrest-specific 6, *AXL* AXL receptor tyrosine kinase/tyrosine-protein kinase receptor UFO, *APOE* apolipoprotein E, *TYROBP* transmembrane immune signalling adaptor TYROBP, *B2M* beta-2-microglobulin, *TREM2* triggering receptor expressed on myeloid cells 2, *IL-1* interleukin-1, *HER2* human epidermal growth factor receptor 2, *CSF1* colony-stimulating factor 1, *SPP1* secreted phosphoprotein 1, *ITGAX* integrin subunit alpha X, *ECM* extracellular matrix, *TLR4* toll-like receptor 4. Adapted from the graphical abstract and Figure 7F in [52], with permission from Elsevier

TAMs also contribute to ECM remodelling in brain metastases, further underscoring their functional diversity. Klemm and colleagues showed in multiple tumour types that MDMs upregulate both pro- and anti-metastatic ECM-related pathways involving lumican (*LUM*), osteoglycin (*OGN*) and SPARC-like 1 (*SPARCL1*) (Fig. 4; 5) [83]. Other work has reported that melanoma brain metastasis cells induce matrix metalloproteinase 2 (MMP-2) in TAMs and vice versa [73]. MMPs can promote tumour growth and metastasis by cleaving a wide range of substrates, including growth factor-binding proteins and structural ECM components [42].

Interestingly, the engagement between TAMs and the ECM also involves reciprocal actions of tumour cells (Fig. 5) [52]. In brain metastasis mouse models of both triple-negative and HER2+ breast cancer, these subtypes showed distinct colonising architectures after metastatic seeding [52]. Triple-negative cells form perivascular sheaths across a wide surface area (Fig. 5; 1a), whereas HER2+ cells grow as spheroid clusters from discrete points on the microvasculature (Fig. 5; 2a) [52]. In triple-negative cells, this increases access to pro-survival growth arrest-specific 6 (GAS6)–tyrosine-protein kinase receptor UFO (AXL) signalling (Fig. 5; 1b) [52]. HER2+ cells promote this method of growth by remodelling the ECM through deposition of tenascin-C (TNC) (Fig. 5; 2b) [52]. This is thought to prevent phagocytic AXL+ microglia from infiltrating the growing tumour [52]. Paradoxically, the phagocytic phenotype of microglia in this context appears to be induced by TNC—Toll-like receptor 4 (TLR4) binding and activation [52]. The spheroid growth pattern may restrict microglia to the tumour periphery and provide a compensatory response to the TNC-originating phagocytic phenotype [52]. TNC suppression prevents metastatic colony development in HER2+ tumours [52]. While this presents a therapeutic opportunity, such targeting may hamper the anti-tumour phagocytic capacity of microglia over time. Therapeutic exploitation should take this into account and may require combinatorial strategies to overcome.

Taken together, recent studies have highlighted that TAMs play both pro- and anti-tumour roles in brain metastases. Functionality ranges from predominantly phagocytic to immunosuppressive states in a context-dependent manner. TAMs also reciprocally remodel the ECM in a tumour subtype and architecture-specific way, which has implications for how experimental therapies should be directed within this framework. Despite this diversity, most evidence indicates that TAMs undergo time-dependent changes towards increasingly pro-tumoural phenotypes. This transition arc seems to occur across tumour types and could be a promising target for future therapies.

## Peripheral immune cells

Peripheral immune cell infiltration into the brain is normally limited by the BBB [11]. In brain metastasis, BBB disruption facilitates the entry of macrophages, neutrophils and lymphocytes. The contribution of these infiltrating cells to both tumour suppression and progression has been a topic of keen interest in recent years.

### Neutrophils

**Fig. 6:**
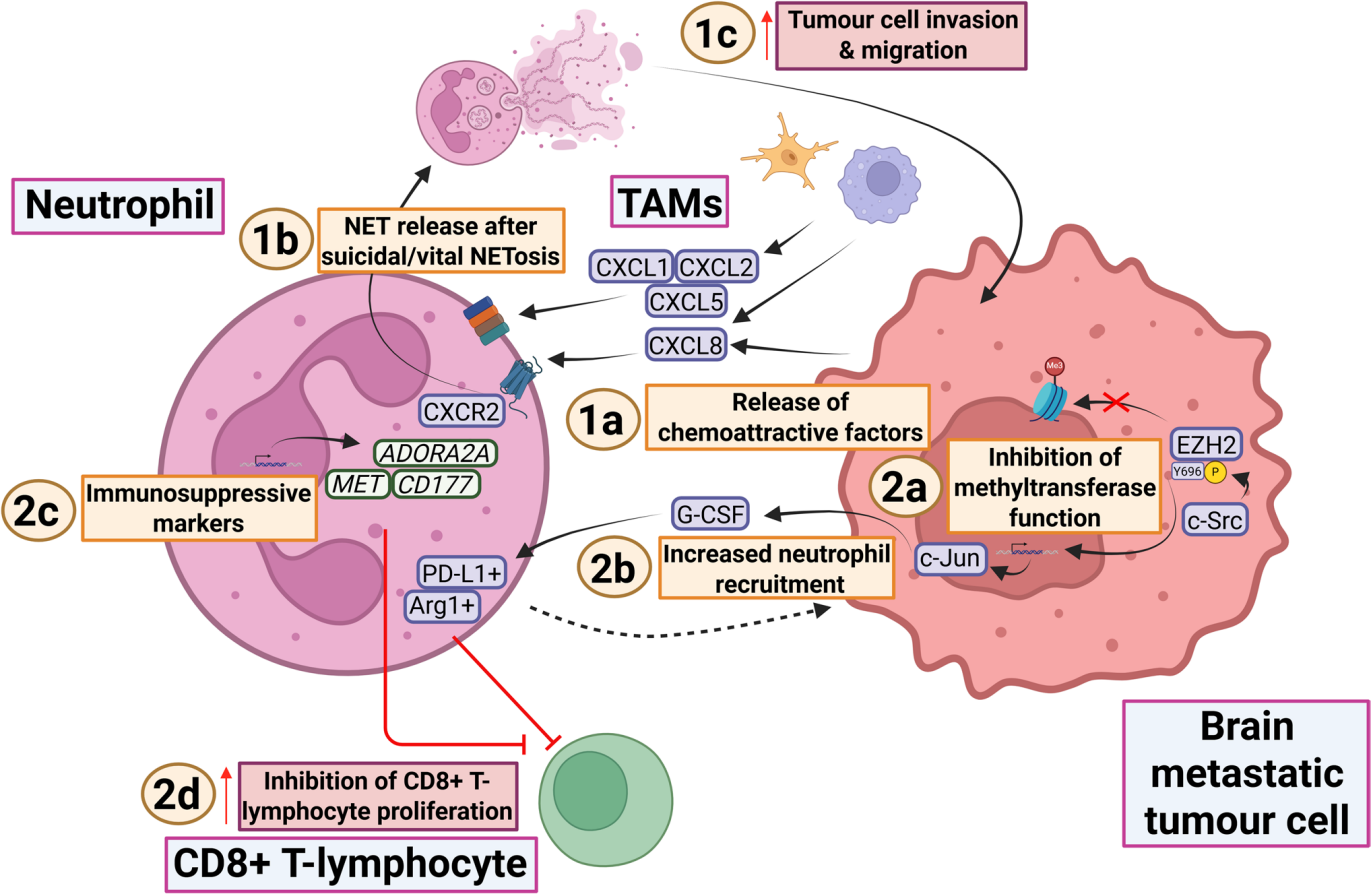
Neutrophils engage in tumour-promoting activity through multiple mechanisms. **1a** Mediators of neutrophil chemoattraction and recruitment to brain metastatic tumour cells include CXCL8 (IL-8), CXCL1, CXCL2 and CXCL5 (original evidence for some mediators at mRNA levels alone, but depicted here as protein complexes for clarity). **1b** CXCL8 (IL-8) binds its receptor, CXCR2, triggering the release of NETs, which can occur after both suicidal or vital NETosis. **1c** NET release has been shown to promote tumour cell invasion and migration. This may relate to the neutrophils adopting a unique spatial distribution within the TME due to their pro-NETotic state, although the underlying mechanisms remain unclear. **2a** EZH2 canonically functions as a histone methyltransferase, but in brain metastatic tumour cells EZH2 is phosphorylated at an alternative tyrosine (Y)-696 binding site by c-Src, altering its activity. **2b** This alternative phosphorylation changes the function of EZH2 to bind RNA polymerase II, leading to upregulation of c-Jun and the recruitment of PD-L1+/Arg1+ neutrophils via G-CSF. **2c** In addition to PD-L1 and Arg1, other markers of immunosuppressive neutrophils in brain metastases include *ADORA2A*, *MET* and *CD177*. **2d** Immunosuppressive neutrophils assist in tumour outgrowth by inhibiting the proliferation of CD8+ T-lymphocytes. Figure created with BioRender (Toronto, Ontario, Canada) Govaerts, C. (2026) https://BioRender.com/c82t538 is licensed under CC BY 4.0. Note: the pathway involving EZH2 and c-Src has also been illustrated in a figure of a prior review [47]. Colour coding— brown/beige: numbered headings; purple/blue: proteins or protein complexes; green: genes indicating mRNA expression; orange: descriptions of mechanisms; crimson: descriptions of pro-tumour effects; pink: cell types. *TAMs* tumour-associated macrophages, *CXCL8/IL-8* chemokine (C–X–C motif) ligand 8, *CXCR2* interleukin 8 receptor, beta, *NET* neutrophil extracellular trap, *c-Src* proto-oncogene c-Src, *EZH2* enhancer of zeste homolog 2, *c-Jun* transcription factor jun, *G-CSF* granulocyte colony-stimulating factor, *PD-L1* programmed death-ligand 1, *Arg1* arginase-1, *ADORA2A* adenosine A2a receptor, *MET* MET protooncogene, receptor tyrosine kinase, *CD177* cluster of differentiation 177 [47, 78, 83, 100, 131, 191, 193]

Neutrophils undergo systemic expansion in response to tumour formation [32]. Their contribution to tumour progression has been overlooked even though neutrophil participation in metastasis was postulated already in the late 1980s [32, 71, 178]. Recent studies now reveal the diverse activation states of neutrophils in brain metastasis. In this section, their potential use as peripheral biomarkers is discussed, as well as the mechanisms regulating their recruitment and intratumoural activity (Fig. 6).

#### ‘Neutrophil-to-lymphocyte ratio’ as a biomarker

The neutrophil-to-lymphocyte ratio (NLR), determined by routine peripheral blood measurements, has predictive value for clinical outcome in brain metastases. An elevated NLR is thought to reflect systemic inflammation that favours cancer cell proliferation and metastasis [59]. Higher NLR values, both pre- and post-treatment, have been associated with worse outcomes in brain metastasis patients undergoing stereotactic radiosurgery [29, 30, 192]. In NSCLC, a high NLR is a predictor for the development of brain metastasis [86]. Beyond its prognostic value, the potential utility of the NLR lies in its affordability and broad applicability. It is, however, unlikely that the NLR reflects a CNS-specific tumour-promoting mechanism, as similar correlations have been made in extracranial cancers [34].

#### The recruitment of tumour-promoting neutrophils

Neutrophils are a prominent component of the infiltrating leucocyte population in brain metastasis, particularly compared with gliomas [9, 83, 100]. Spatial transcriptomic, mRNA-deconvolution, multiplex immunofluorescent imaging and mass-cytometry approaches show that neutrophils localise primarily to the perivascular niche within tumours [77, 100, 193]. TAM cell expression of *CXCL8* (IL-8), *CXCL1*, *CXCL2* and *CXCL5* promotes neutrophil chemoattraction in brain metastases from various tumour types (Fig. 6; 1a) [83, 100]. IL-8, produced by NSCLC and gastrointestinal brain metastatic tumour cells, also recruits neutrophils [78, 193]. Corroborating this, neutrophils upregulate the IL-8 receptor C–X–C motif chemokine receptor 2 (*CXCR2*) after exposure to breast cancer brain metastasis cells (Fig. 6; 1b) [131]. CXCR2 signalling enhances neutrophil infiltration specifically to peripheral stromal areas and promotes neutrophil extracellular trap (NET) formation and release in a process called NETosis (Fig. 6; 1c) [131]. This ‘pro-NETotic’ neutrophil state driven by CXCR2 may facilitate the migration and invasion of metastatic cells at the tumour periphery [131].

These observations provide a foundation for understanding the factors involved in neutrophil recruitment. The precise cellular processes underlying tumour-promoting neutrophil chemoattraction are, however, poorly understood. A novel mechanism was recently discovered involving enhancer of zeste homolog 2 (EZH2) (Fig. 6; 2a) [191]. In brain metastases, the canonical function of EZH2, trimethylation of histone H3K27, can be suppressed through phosphorylation by proto-oncogene c-Src (c-Src) [191]. Phosphorylated EZH2 subsequently binds RNA polymerase II, leading to transcriptional upregulation of Jun proto-oncogene, AP-1 transcription factor subunit (*JUN*) and resulting in granulocyte (G)-CSF-dependent recruitment of arginase-1 (Arg1)+/PD-L1+ neutrophils (Fig. 6; 2b) [191]. This correlates with brain metastasis outgrowth, primarily by suppressing the proliferation of activated CD8+ T-lymphocytes [191]. In sum, neutrophils infiltrate brain metastases via multiple cyto- and chemokines, notably IL-8 and G-CSF. The underlying mechanisms are not well understood, but include EZH2-driven *JUN* upregulation in tumour cells.

#### Diverse functions and phenotypes of neutrophils in brain metastasis

Recruited neutrophils in brain metastases are characterised by high expression of Adenosine A2a Receptor (*ADORA2A*), CD177 molecule (*CD177*) and MET proto-oncogene receptor tyrosine kinase (*MET*) (Fig. 6; 2c) [83]. These markers are associated with immunosuppressive neutrophils and have been linked to polarisation towards a pro-tumourigenic ‘N2’-like phenotype that suppresses T-lymphocyte proliferation (Fig. 6; 2d) [55, 98, 185].

Although neutrophils are often immunosuppressive, recent findings indicate that their transcriptomes are highly heterogeneous and can be enriched for multiple pro-inflammatory pathways [100]. Multiparametric immunogenomic analyses have shown that neutrophil phenotypes are highly variable. For instance, scRNA-seq analysis of resected brain metastasis material from melanoma patients identified three distinct mature neutrophil clusters [8]. The first was a ‘calprotectin-high’ group, characterised by expression of *S100A8* and *S100A9*, which encode the calprotectin heterodimer [8]. The second comprised neutrophils with elevated expression of genes linked to the IFN response, while a third cluster showed activity related to IL-8 signalling [8]. Gene expression within the IL-8-high group corresponded most to a classical pro-tumour N2-like signature and was associated with angiogenesis and tumoural epithelial-to-mesenchymal transition (EMT) [8].

Neutrophil activity is also highly context-dependent and affected by both the primary tumour type and the genomic profile of the metastatic lesion [9]. Kirsten rat sarcoma virus (*KRAS*) and tumour protein p53 (*TP53*)-mutant (*TP53*^*mut*^ and *KRAS*^*mut*^) lung brain metastases are more heavily infiltrated by neutrophils than are non-*TP53*^*mut*^ or *KRAS*^*mut*^ tumours [9]. Neutrophils in *TP53*^*mut*^ lesions exhibit a distinct transcriptomic signature indicative of metabolic reprogramming towards mitochondrial fatty acid oxidation, facilitating reactive oxygen species (ROS) release and promoting an immunosuppressive phenotype [9]. Furthermore, neutrophils appear to play a distinct role in breast cancer brain metastases with genomes characterised by high levels of focal hypermutation, a phenomenon known as ‘kataegis’ [9]. In contrast to non-kataegic breast brain metastases, neutrophils in kataegic lesions display pro-inflammatory features, indicated by enrichment for IFN response and degranulation-linked genes [9].

Overall, neutrophils are abundant in the TME of brain metastases, yet their apparently multifaceted and dynamic roles remain poorly understood. Future work should focus on a better classification of neutrophil phenotypes and on elucidating their context-dependent pro- and anti-inflammatory activities. Increased mechanistic understanding of how distinct neutrophil subgroups interact with and modulate CD8+ T-lymphocytes will be critical, as this will likely influence immune therapy responses. Such insights may guide the development of novel combinatorial therapies.

### T- and B-lymphocytes

CD3- and particularly CD8+ T-lymphocytes are crucial mediators of anti-tumour immunity [21]. Early assessments of lymphocyte infiltration in brain metastases showed that they localise primarily to interstitial and perivascular areas between dense tumour clusters [132, 157]. Comparisons with gliomas in those studies generally aligned with the now widely recognised observation that lymphocyte infiltration occurs at higher levels in brain metastases [51, 77, 83, 132, 157]. This high rate of T-lymphocyte influx may explain the greater sensitivity of brain metastases to ICI-therapy compared to gliomas [40, 43, 56, 66, 85, 120, 121, 138, 161, 165, 169].

**Fig. 7:**
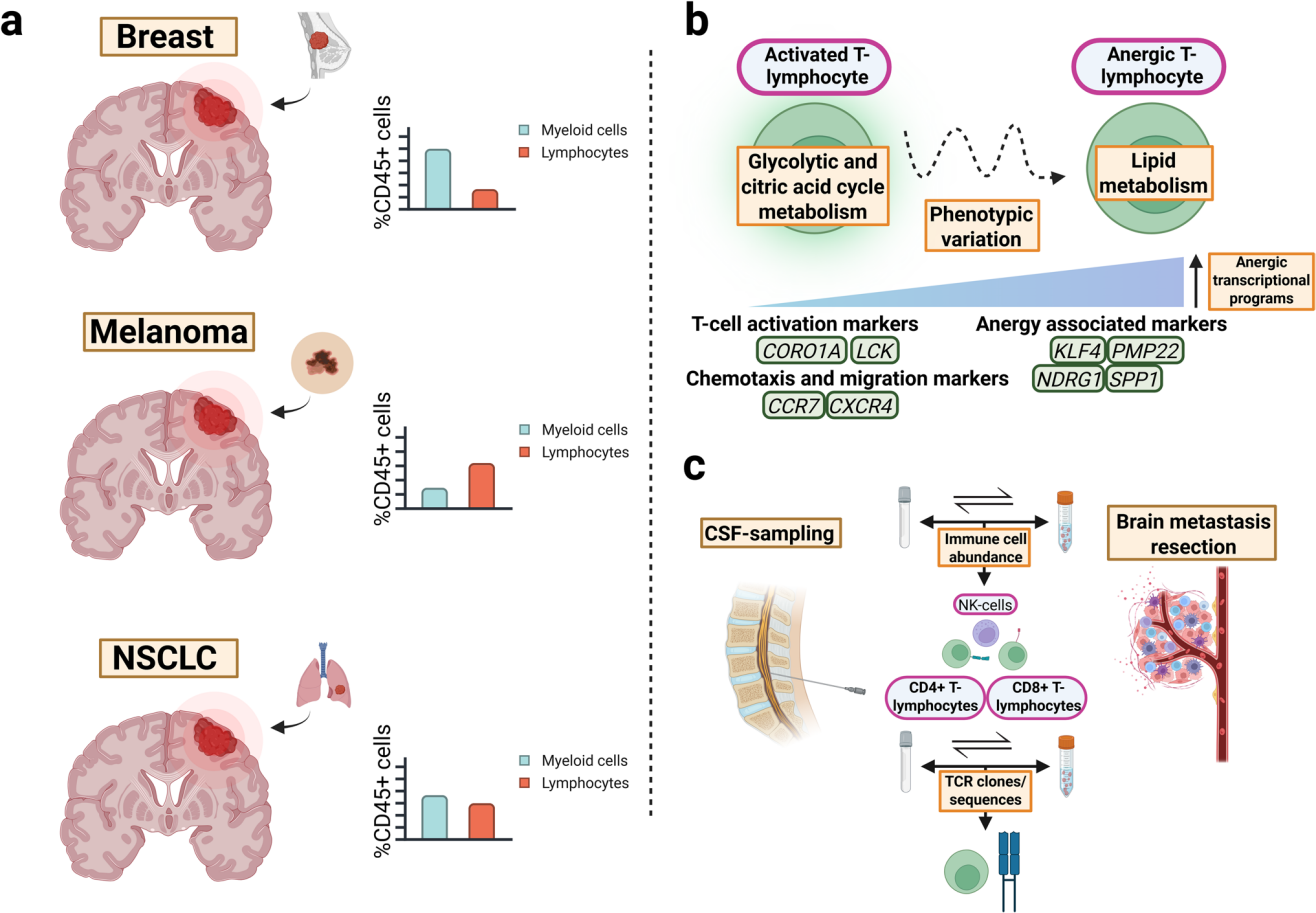
The dynamics of tumour-infiltrating lymphocytes in brain metastasis. **a** The proportion of lymphocytes among tumour-infiltrating CD45+ cells differs depending on the primary tumour type in brain metastases. Bar charts show these relative values by tumour type, with light blue indicating myeloid cells and red indicating lymphocytes. The column values are based on estimates derived from the graphs in Figure 1F by Klemm and colleagues [83], and are additionally based on observations from other studies cited below. **b** Single-cell sequencing data from Gonzalez and colleagues [57] indicate that lymphocyte functional inactivity and anergy in brain metastases correspond to a metabolic shift from glycolysis to lipid metabolism. Gene expression markers associated with anergy include *KLF4*, *PMP22*, *NDRG1* and *SPP1*. Diffusion maps of lymphocyte clusters show that phenotypic variation is largely driven by T-lymphocyte activation markers such as *CORO1A* and *LCK*. Further variation between clusters is seen in chemotaxis and migration, with differential expression of markers such as *CCR7* and *CXCR4*. **c** Using direct CSF sampling and tumour digestion, Rubio-Perez and colleagues showed that the abundance of NK cells, CD4+ T-lymphocytes and CD8+ T-lymphocytes is comparable between the TME and the CSF [130]. Notably, TCR sequences are also largely consistent between these two compartments. These findings suggest that lumbar puncture could serve as a relatively non-invasive means of assessing the intracranial immune landscape and identifying suitable candidates for immune therapy. Figure created with BioRender (Toronto, Ontario, Canada). Govaerts, C. (2026) https://BioRender.com/l84b537 licensed under CC BY 4.0. Colour coding—brown/beige: headings; green: genes indicating mRNA expression; orange: descriptions of mechanisms/subheadings; pink: cell type. *NSCLC* non-small cell lung cancer, *CD45* cluster of differentiation 45, *CSF* cerebrospinal fluid, *NK* natural killer, *CD4* cluster of differentiation 4, *CD8* cluster of differentiation 8, *TCR* T-cell receptor, *CORO1A* coronin 1A, *LCK* LCK proto-oncogene, *Src* family tyrosine kinase, *CCR7* CC motif chemokine receptor 7, *CXCR4* CXC motif chemokine receptor 4, *KLF4* Krüppel-like factor transcription factor 4, *PMP22* peripheral myelin protein 22, *NDRG1* N-Myc downstream regulated 1, *SPP1* secreted phosphoprotein 1, *TME* tumour microenvironment [13, 14, 38, 51, 57, 64, 65, 77, 80, 83, 130, 159, 175, 183]

More recent work has provided detailed insights into the extent of lymphocyte infiltration in brain metastases, which are best appreciated separately by tumour type (Fig. 7a).

#### Quantification, localisation and prognostic value of tumour-infiltrating lymphocytes

The breast cancer brain metastasis TME contains relatively fewer CD3/CD8+ T-lymphocytes compared to metastases from other primary tumours [65, 77, 83, 183]. Numbers of CD4+ and CD8+ T-lymphocytes are decreased in the brain compared to primary breast tumours [31, 136, 149]. The receptor status of the breast cancer brain metastasis also influences lymphocyte infiltration. For example, triple-negative tumours have a higher percentage of cytotoxic CD8+ T-lymphocytes compared to HER2+ tumours, and hormone receptor (HR)-negative tumours have fewer numbers of PD-L1-positive tumour cells interacting with T-lymphocytes than HER2-/HR+ tumours [60]. High infiltration of CD4+ and CD8+ T-lymphocytes in breast cancer brain metastases has been found to correlate with improved overall survival, although this may be subtype dependent [33, 60, 149]. Prognostic value is also determined by relative immune composition, as a low stromal CD4+/CD8+ lymphocyte ratio predicts favourable outcomes [60]. In addition, greater density of intratumoural tertiary lymphoid structures correlates positively with overall survival, as has been shown in various treatment contexts for systemic extracranial cancers [23, 69, 198]. Tertiary lymphoid structures are organised aggregates of lymphoid cells that arise in pathological states [197]. They are thought to be able to facilitate a more effective local immune response, explaining this positive prognostic association [23, 69, 197].

On the other hand, CD3+ and CD8+ T-lymphocyte infiltration is relatively prominent in melanoma brain metastases [13, 64, 65, 77, 83, 183]. Among primary tumours, the TME of melanoma appears the most “immune-hot”, which is reflected by an abundance of CD8+ T-lymphocytes and an elevated CD8+ /CD3+ lymphocyte ratio [14, 51, 65, 77, 83, 183]. The brain is the least lymphocyte infiltrated metastatic site in melanoma, followed by melanoma skin metastases distant from the original tumour [84, 177]. CD3+, CD8+ and PD-1+ lymphocyte populations have a spatial and a strong statistical correlation with PD-L1 expression in brain metastatic melanoma cells, indicating an immunologically active yet exhausted TME [13, 65]. This could explain the relative success seen with ICIs in melanoma brain metastases [85, 161]. Prognostic associations of CD3+ and CD8+ lymphocyte infiltration, as well as their ratios, remain inconsistent, with some studies reporting favourable prognostic assessments [64, 84] and others none [13, 65].

In NSCLC brain metastases, T-lymphocyte density is intermediate to breast cancer and melanoma [65, 77, 83, 183]. Similar to other cancers, CD8+ T-lymphocyte infiltration is decreased in the brain compared to extracranial metastatic sites, although results are variable [159, 175]. One study reported a non-significant decrease of T-lymphocytes in the brain [80], whereas another showed a significant reduction only when comparing deeply infiltrating cells [91]. Prognostic associations are also varied, with one study reporting that deep CD4+ and CD8+ T-lymphocyte infiltration alone predicts for favourable outcomes [118]. This compares with other findings of no significant prognostic association when tumours are stratified by stromal lymphocyte infiltration, while a marked association is observed in tumours with a prominent peritumoural infiltrate [162]. On the other hand, increased CD3+ lymphocyte infiltration has been associated with worse prognosis in some cohorts [80]. Similar to breast cancer brain metastases, the number of tertiary lymphoid structures intratumourally does seem to have prognostic benefit [113]. These observations indicate that spatial and structural patterns of lymphoid organisation in tumours should be considered in addition to quantifications of immune infiltration. PD-L1 positivity also appears to predict clinical outcome, as one study found a positive prognostic correlation only when combining PD-L1 positivity with lymphocyte infiltration in NSCLC brain metastases [91]. This is also reflected in the positive objective response rates to pembrolizumab when stratifying NSCLC brain metastasis patients by PD-L1 expression [56].

In summary, T-lymphocyte infiltration is highest in melanoma, followed by NSCLC and breast cancer (Fig. 7a). A more detailed understanding of their comparative lymphocyte landscape, incorporating spatial and temporal dynamics, may help explain differences in ICI therapy response across these tumour types.

#### Phenotypic heterogeneity of lymphocytes

scRNA-seq analyses have largely validated that brain metastases of different tumour types differ in terms of immune composition and that compared to primary lesions, brain metastases are more deficient in activated, differentiated and effector memory T-lymphocytes [159]. The marked diversity in T-lymphocyte functional states across various brain metastasis tumour types has also been clarified through single-cell profiling techniques. One study analysing 15 separate brain lesions identified multiple clusters of CD8+ effector memory T-lymphocytes, CD4+ central memory T-lymphocytes and regulatory T-lymphocytes [57]. Diffusion maps indicated that variation in lymphocyte activation, driven by coronin 1A (*CORO1A*) and the LCK proto-oncogene, Src family tyrosine kinase (*LCK*), was a major determinant of phenotypic variation between lymphocyte clusters [57]. In addition, differences in chemotaxis and migration were observed, likely mediated by C–C motif chemokine receptor 7 (*CCR7*) and *CXCR4* [57]. Several clusters were also functionally inactive, evidenced by the upregulation of anergy-associated gene programmes [57]. This inactivity was accompanied by a shift from glycolysis to predominantly lipid metabolism [57] (Fig. 7b). Lipids are recognised as crucial signalling molecules in dictating T-lymphocyte functioning [93]. Similar examples of metabolic rewiring have been reported to lead directly to the impairment of effector T-lymphocyte processes in various tumour types [57, 170].

#### Tumoural and molecular determinants of T-lymphocyte functioning

Separate from the broad phenotypic heterogeneity of T-lymphocytes in brain metastases, emerging work is starting to define the determinants of T-lymphocyte state and function. The nature and degree of difference of the effector and exhausted states of T-lymphocyte populations between primary and brain metastatic lesions depend on the primary tumour type [183]. For example, of all tumour types T-lymphocytes in brain metastatic colorectal tumours seem to show the largest reduction in effector function compared to lymphocytes found in primary colorectal tumours [183]. T-lymphocytes from melanoma brain metastases on the other hand are generally more exhausted than their systemic counterparts [183]. Collating these observations into a single working model of T-lymphocyte reprogramming in brain metastases in a tumour-type-specific manner may help explain discordant responses to ICI therapy seen between intracranial and extracranial lesions.

T-lymphocyte function and phenotype are not only regulated by tumour histology but also by tumour-intrinsic genomic features, similar to what was reported in neutrophils. Álvarez-Prado and colleagues demonstrated that in breast and lung brain metastases, respectively, kataegic and *TP53*^*mut*^ tumours are more enriched for functionally active T-lymphocytes than non-*TP53*^*mut*^ and non-kataegic lesions [9]. The contribution of spatial niches to T-lymphocyte dynamics has also received attention. Spatial immunogenomic profiling found that the TCR repertoire in glioblastoma is more heterogeneous than in brain metastases [134]. This was evidenced by a higher level of spatial restriction of specific clonal variants present within defined regions and a greater inter-regional repertoire diversity [134]. This suggests that extrinsic stimuli such as inflammatory cytokines, which promote clonal expansion, are less spatially confined in brain metastases than in glioblastoma [134]. This provides another explanation for the contrasting ICI-response rates seen between brain metastases and gliomas, as the increased spatial complexity in the latter could be driving more divergent T-lymphocyte responses.

#### Cerebrospinal fluid as a surrogate for T-lymphocyte profiling

Recent efforts have shown that the immune landscape in brain metastases across multiple tumour types closely correlates with the immune profile observed in the cerebrospinal fluid (CSF), providing a potential clinical application [130] (Fig. 7c). Rubio-Perez and colleagues showed that quantities of CD8+ and CD4+ T-lymphocytes and natural killer (NK) cells are comparable between the CSF and TME, and importantly, TCR sequences appear to be largely maintained [130]. Subsequent studies comparing the brain metastasis immune compartment within the CSF have further confirmed these observations [92, 199]. The strong similarity between the brain metastatic TME and CSF immune landscapes suggests that lumbar puncture could serve as a minimally invasive method to identify candidates for ICI-therapy or to allow for TCR-guided approaches to other immune-based treatments [130].

#### B-lymphocytes

B-lymphocytes are far less abundant than T-lymphocytes in the brain metastasis TME and their role is less well understood [149]. A study reported that brain metastasis patients with prolonged overall survival and minimal rates of intracranial relapse after resection and stereotactic radiosurgery had tumours enriched for B-lymphocyte-related transcripts [151]. Follow-up immunohistochemistry identified that infiltration by CD138 plasma cells, representing effector B-lymphocytes, is a favourable prognostic factor [151]. More recently, murine models of brain metastases showed that recruitment of B-lymphocytes by specific subpopulations of M1-like macrophages is required for an effective response to combined CTLA-4/PD-1 blockade [112]. Although the full implications are not yet clear, these findings suggest that appropriate B-lymphocyte coordination, similar as in extracranial cancers, is important for intracranial ICI-efficacy [26, 171]. Accordingly, scRNA-seq studies indicate that brain metastatic tumours are often more enriched for B and plasma cells than primary tumours of the same histology [16, 183]. Spatial transcriptomic analyses have also indicated that B-lymphocytes form aggregates in compartmentalised regions with brain metastases, perhaps reflecting tertiary lymphoid structures [16, 159]. Tumours with high numbers of these structures could represent suitable candidates for ICI therapy [16, 23].

To summarise, current evidence indicates that quantification of tumour-infiltrating lymphocytes has prognostic value in brain metastases from the three most prominent tumour types: NSCLC, breast cancer and melanoma. Other findings, such as the observation in NSCLC that lymphocyte spatial orientation and organisation associate with prognosis, emphasise the need to further develop methodology for assessing immune cell infiltration. Additional prospective investigations at the single-cell level are urgently needed to understand how lymphocyte populations and TCR-repertoires evolve in response to ICI therapy and how these dynamics correlate to specific spatial niches. In particular, identifying the differences that define brain metastatic lesions with divergent responses is a key priority and could help optimise treatment strategies and improve patient selection for therapy.

### Natural killer cells

NK cells are cytotoxic lymphocytes that promote the lysis or apoptosis of both infected and tumour cells. They show increased efficacy against those specifically lacking MHC-1 expression [181]. They act in a complementary manner to CD8+ T-lymphocytes and rely for their activation on interactions like those between the natural killer group 2D receptor (NKG2DR) and its ligands [46].

Little is understood of the dynamics of NK cells in brain metastases and they likely constitute only a minor proportion of the total lymphocyte compartment [77, 83, 148, 176]. Similar to CD8 and CD4+ T-lymphocytes, however, they may infiltrate brain metastases more effectively than gliomas and appear to adopt distinct phenotypes [77]. Recent work has attempted to characterise NK-cell subpopulations. Friebel and colleagues used single-cell mass cytometry in gliomas and brain metastases from various primary sites to separate two putative NK-cell populations, CD56^int/bright^CD16^−^ and CD56^int^CD16+ [51]. These two clusters corresponded to immature and cytotoxic NK cells, respectively [51, 146]. Brain metastases were enriched for CD56^int^CD16+ cytotoxic cells, which highly expressed CD244, CD38 and antigen-kiel 67 (Ki-67) [51]. Isocitrate dehydrogenase (IDH) wildtype (WT) gliomas on the other hand showed a greater abundance of CD56^int/bright^CD16^−^ immature NK cells [51]. The increased presence of cytotoxic NK cells in the TME of brain metastases may partially contribute to their better response rates to ICI-therapy compared to gliomas. This link was recently proposed elsewhere [48, 87]. Using a murine melanoma brain metastasis model, it was shown that combined PD-1/CTLA-4 blockade increases CD8+ T-lymphocyte infiltration primarily via enhanced cell trafficking [48]. This seemed to be dependent on NK-cell production of chemokines such as CXCL9 and CXCL16 [48]. This implies that in understanding the mediators of sustained ICI therapy responses in brain metastases, more emphasis should be placed on the role of NK cells.

These observations also raise the question of how NK cells can be leveraged for therapeutic purposes in brain metastases. One vulnerability involves the localisation of NKG2D ligands (NKG2DL), which are stably expressed in brain metastases and co-localise with a subset of breast and lung tumour cells exhibiting markers of cellular dormancy [49]. This indicates that enhancement of NKG2DR-NKG2DL-mediated cell killing may provide a therapeutic approach to target quiescent subclones and reduce the risk of recurrence. In other work, targeted NK-92 cells were delivered to the brain in xenografted HER2 expressing breast brain metastasis models [6, 7]. The NK-92 cells were virally transduced to express a HER2 chimeric antigen receptor [6, 7]. After systemic injection, their intracranial entry was promoted with BBB disruption using MRI-guided focussed ultrasound (FUS) [6]. NK-cell therapy combined with FUS was more effective in reducing tumour volume and improving overall survival compared to either FUS or cell therapy alone [6].

Taken together, although NK cells have only recently been studied in brain metastases, current results show that they exert important functions and can enhance CD8+ T-lymphocyte activity. Future work should determine whether therapeutic NK-cell-based approaches provide additional benefit when applied in combination with existing immune-based therapies.

## Understudied cell types

A notable understudied brain-resident cell type in the context of brain metastases are oligodendrocytes. In gliomas, oligodendrocyte progenitor cells have been implicated in inducing stemness and chemo-radioresistance at the tumour border, potentially contributing to tumour recurrence after resection and adjuvant therapy [67]. This suggests that oligodendrocytes and their precursors also have the potential for tumour-modulating activity in brain metastases. Unfortunately, their role in this context is understudied, emphasising the need for further research to understand their contribution to the brain metastasis TME.

Another comparatively understudied cell type is cancer-associated fibroblasts. Several recent publications suggest that they have tumour-modulating roles in brain metastases and that their contribution to malignancy should be studied more widely [3, 5, 58].

## Concluding remarks

**Fig. 8:**
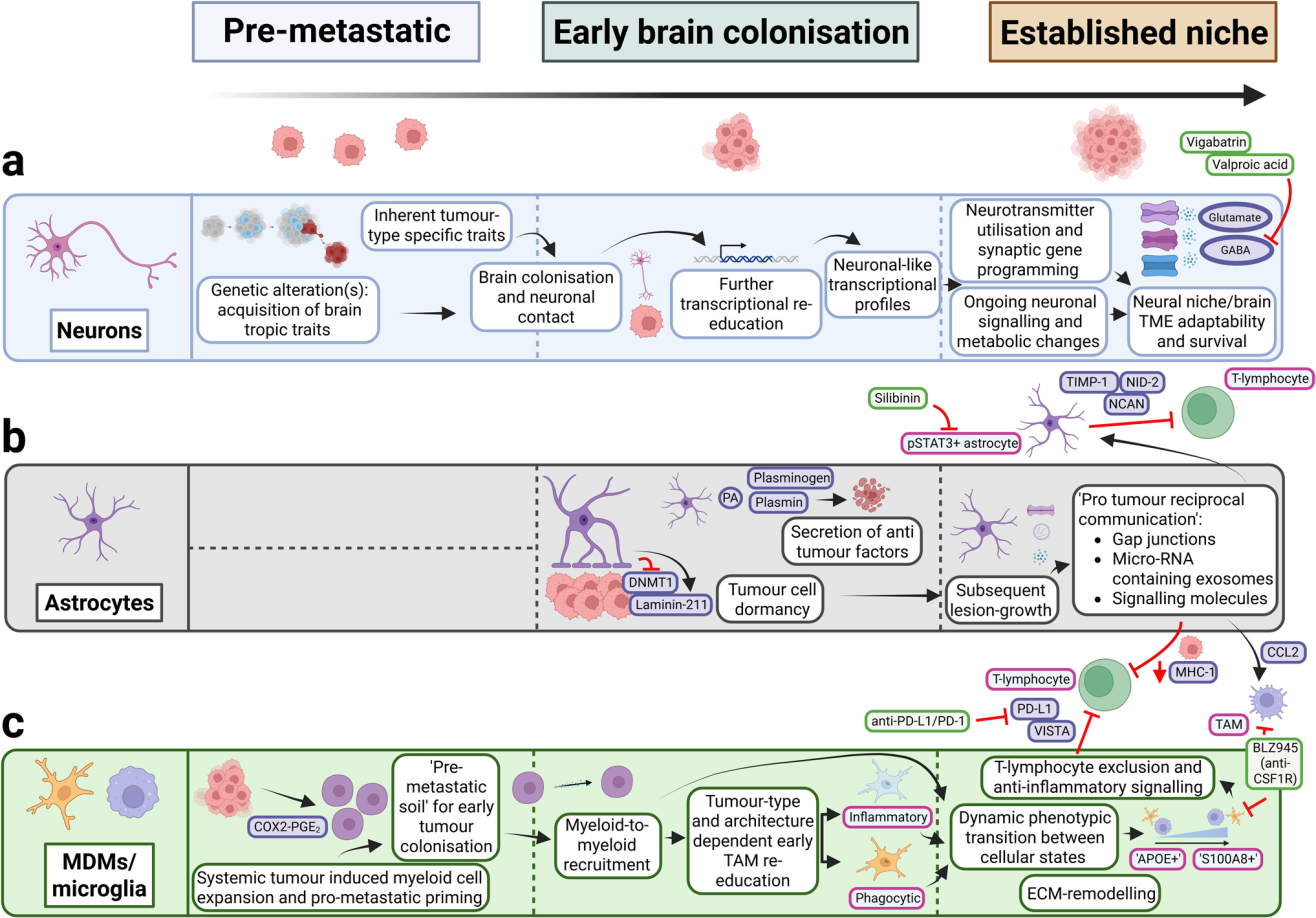
Microenvironmental contributions to stepwise brain metastatic progression and therapeutic opportunities. **a** Neural rewiring and signalling contribute to metastatic progression in brain metastases. Tumour cells acquire brain tropic traits through genetic alterations, which along with tumour-type-specific features such as those seen in melanoma increase the probability for successful brain colonisation. Subsequent neuronal contact further reprograms tumour cells to develop ‘neuronal-like’ states, as evidenced by the upregulation of genes linked to synaptic activity, development and plasticity. These changes allow tumour cells to exploit the neurotransmitter-rich milieu of the brain. Together with ongoing neuronal signalling and pro-tumour metabolic changes, this provides tumour cells with the machinery to adapt and survive within neural niches in the brain TME. **b** Astrocytes have various roles throughout the metastatic process. They adopt diverse pro- and anti-tumour functions upon tumour cell extravasation. These include the secretion of tumour suppressive factors like PA but also the induction of tumour cell dormancy. Dormancy enables long-term tumour cell survival and subsequent tumour outgrowth under permissive conditions. In established lesions, astrocyte–tumour interaction enhances tumour growth by communication via gap junctions, microRNA containing exosomes and various secreted signalling molecules. Astrocytes also shape the immune landscape. Examples of this include pSTAT3+ astrocytes suppressing T-lymphocyte activity through factors such as TIMP-1, the reduction of MHC-1 expression on tumour cells and the recruitment of anti-inflammatory TAMs via CCL2 signalling. **c** MDMs/microglia play key roles in brain metastasis development. Prior to brain colonisation, systemic tumours drive COX2-PGE_2_-mediated expansion of immature myeloid cells, which enter the brain and establish a tumour-permissive ‘premetastatic soil/niche’ that facilitates brain colonisation. TAMs are re-educated towards inflammatory and phagocytic phenotypes in a tumour type- and architecture-dependent manner. In established lesions, TAMs engage in ECM remodelling and show remarkable phenotypic diversity as shown through scRNA-seq. TAMs have been identified as ‘APOE+’ and ‘S100A8+’ subsets, which correlate with pro- and anti-inflammatory functionality, respectively. Anti-inflammatory TAMs cause T-lymphocyte exclusion through the upregulation of specific markers including PD-L1 and VISTA. Figure created with BioRender (Toronto, Ontario, Canada). Govaerts, C. (2026) https://BioRender.com/licv9ya is licensed under CC BY 4.0. Colour coding—light blue: neuron-specific processes; black: astrocyte-specific processes; green: MDM/microglia-specific processes; purple/blue: proteins or protein complexes; pink: other cell types; light green: therapeutic agents. *GABA* gamma-aminobutyric acid, *DNMT1* DNA methyltransferase 1, *PA* plasminogen activator, *(p)STAT3* (phosphorylated) signal transducer and activator of transcription 3, *TIMP-1* tissue inhibitor of metalloproteinase (TIMP) metallopeptidase inhibitor 1, *NID-2* nidogen-2, *NCAN* neurocan, *MHC-1* major histocompatibility complex 1, *MDM* monocyte-derived macrophage, *COX2* cyclooxygenase-2, *PGE*_*2*_ prostaglandin E2, *TAM* tumour-associated macrophage, *ECM* extracellular matrix, *APOE* apolipoprotein E, *S100* S100 calcium-binding protein, *PD-L1* programmed death-ligand 1, *VISTA* V-domain Ig suppressor of T-cell activation, *CSF1R* colony-stimulating factor 1 receptor, *CCL5* chemokine (C–C motif) ligand 5, *scRNA-seq* single-cell RNA sequencing

Our understanding of the cellular landscape of the brain metastasis TME has advanced considerably in recent years. However, delineating the individual components and elucidating their interactions that affect tumour progression remain challenging. This is largely due to the profound heterogeneity both within single lesions and across different tumours, which is reflected in the variable responses of brain metastases to systemic treatment. This review aimed to clarify the underlying sources of this heterogeneity by outlining a working model of the cellular processes shaping the brain metastasis TME (Fig. 8). Different brain-resident cells and peripheral immune players were discussed separately, offering a mechanistic, cell-type-specific overview of their roles in tumour progression. While the focus of this review was on how TME cells promote the malignant potential of tumour cells, a key theme that emerged throughout is the functional diversity of the cellular TME and the lack of a clearly defined pro- or anti-tumour role for any single-cell type. A good example of this is in TAM–tumour cell interactions, which appear to transition from primarily tumour suppressing during initial colonisation to tumour enabling as a lesion becomes established. This functional diversity and progressive re-education reflects profound cellular plasticity driven by both tumour-intrinsic and extrinsic stressors. Moreover, inter-tumour heterogeneity is further amplified by the fact that brain metastases are not a single entity but rather encompass a wide range of tumour types. For example, mechanisms observed in breast cancer brain metastases may not necessarily apply to melanoma. Caution is therefore warranted when making general conclusions about cellular dynamics and bi-directional interactions within the brain metastasis TME.

These various challenges require an accelerated use of multi-dimensional approaches to evaluating the architecture of the brain metastasis TME. Integrated scRNA-seq, spatial transcriptomic, proteomic and other genomic approaches, ideally on patient-derived tissues, will provide unprecedented insight into an ecosystem that is difficult to achieve with each method alone. A deeper understanding of temporal dynamics is also required, where tissue sampling at multiple time points should reveal how the TME changes in response to treatment and what features define responders and non-responders. There is an urgent need for future studies to be performed in a tumour-type-specific way that allows for robust comparisons between different variants of primary cancer. Collectively, such efforts should pave the way both for the development of new treatments and the refinement of existing therapies. Ultimately, a future can be envisioned where techniques are developed that evaluate tumour-specific vulnerabilities at the TME level that can subsequently be exploited in a personalised manner.

## Supplementary Information

Below is the link to the electronic supplementary material.

Supplementary file1 (DOCX 23 KB)

## Data Availability

No original data were generated for this study.
