# Supplementary material for: Future directions in the treatment of brain metastases: evaluating the role of cellular players in the tumour microenvironment
Source: Acta Neuropathol. 2026 May 19;151(1):58. doi: 10.1007/s00401-026-03011-8 (PMC13186852; doi:10.1007/s00401-026-03011-8)
Supplement: Supplementary file 1 — Supplementary file1 (DOCX 23 KB) [file 401_2026_3011_MOESM1_ESM.docx]

**Future directions in the treatment of brain metastases: evaluating the role of cellular players in the tumour microenvironment**

Chris W. Govaerts^1^, J. Marc C. van Dijk^2^, Anouk van der Hoorn^3^, Frank A. E. Kruyt^1^

^1^Department of Medical Oncology, University Medical Center Groningen, University of Groningen, Groningen, The Netherlands; C.W. Govaerts ORCID: 0000-0002-5478-7158; F. A. E. Kruyt ORCID: 0000-0002-2445-9380

^2^Department of Neurosurgery, University Medical Center Groningen, University of Groningen, Groningen, The Netherlands; J.M.C. van Dijk ORCID: 0000-0002-0814-5680
^3^Department of Radiology, Medical Imaging Center (MIC), University Medical Center Groningen, University of Groningen, The Netherlands; A. van der Hoorn ORCID: 0000-0003-4649-327X

Corresponding author:

Frank. A. E. Kruyt

Department of Medical Oncology

University Medical Center Groningen

University of Groningen

Hanzeplein 1

9713 GZ Groningen

The Netherlands

Phone: 050-3615531

E-mail address: [f.a.e.kruyt@umcg.nl](mailto:f.a.e.kruyt@umcg.nl)

ORCID: 0000-0002-2445-9380

*Disclosures and declarations:*

Anouk van der Hoorn reports consulting fees from Quantitas Solutions and Servier. The other authors declare no conflicts of interest

*Acknowledgements and funding:*

This work was supported by an MD/PhD grant supplied by the Graduate School of Medical Sciences of the University of Groningen

*Data availability:*

No original data was generated for this study

FILE CAPTION

Supplemental methods: description of search method and search strategy in MEDLINE.

SUPPLEMENTAL METHODS

**Search method**

The MEDLINE database was searched through PubMed with a curated search strategy. The initial search was performed in June of 2022, with the most recent update being in September of 2025. The search strategy was constructed as broadly as possible, including any and all terms, as well as Medical Subject Headings (MeSH) terms, related to cellular and molecular aspects of the brain metastasis microenvironment. Given that this method yielded many possible relevant articles, our approach was first to sort each one under the following broad headings: ‘exclusions’, ‘diagnostics/imaging’, ‘large-scale TME-characterisation’, ‘clinical cohort studies’, ‘clinical trials’ and ‘specific cell-type characterisation’. Studies were initially screened by title and abstract and excluded if they were case reports, editorials/letters, guidelines/protocols, reviews/meta-analyses or considered irrelevant (not corresponding to the previous headings, not brain-metastasis specific or not related to the TME).

Within each heading, the articles were then subcategorised according to the main subject and/or theme of their contents. Articles under the heading ‘specific cell-type characterisation’ were separately organised in categories related to the cell-type of interest (astrocytes, B-cells, cluster of differentiation-4-positive (CD4+) T-cells, CD8+ T-cells, dendritic cells, extracellular matrix (ECM), fibroblasts/stromal cells, granulocytes, mast cells, microglia/monocyte-derived macrophages, neurons, natural killer (NK) cells, oligodendrocytes and vasculature/endothelial cells) and then also accordingly subsorted. Objective criteria for the inclusion from this list of articles in the main body of the review were not used. This is because of the vast heterogeneity in study methods and subject matter in the collected literature. Rather, articles were chosen on the basis of perceived quality and the novelty or importance of their findings. The reference manager EndNote™ 21.1 (Clarivate Plc, London, United Kingdom) was used for library management and study sorting.

**Search strategy**

Concept 1**:** tumour microenvironment

­ *MeSH*:

"Tumor Microenvironment"[Mesh] OR "Immune System"[Mesh] OR "Hematopoietic System"[Mesh] OR "Neuroglia"[Mesh] OR "Connective Tissue Cells"[Mesh] OR "Endothelial Cells"[Mesh] OR "Neurons"[Mesh] OR "Blood-Brain Barrier"[Mesh] OR "Blood Vessels"[Mesh] OR "Neovascularization, Physiologic"[Mesh] OR "Extracellular Space"[Mesh] OR "Immunotherapy"[Mesh] OR "Antineoplastic Agents, Immunological"[Mesh] OR "Immunotherapy, Adoptive"[Mesh] OR "Antibodies, Monoclonal, Humanized"[Mesh] OR "Cancer Vaccines"[Mesh] OR "Angiogenesis Inhibitors"[Mesh] OR "Oncolytic Virotherapy"[Mesh]

*Keywords*:

TME[tiab] OR microenvironment*[tiab] OR “B lymphocyte*”[tiab] OR “B cell*”[tiab] OR “plasma cell*”[tiab] OR “dendritic cell*”[tiab] OR granulocyte*[tiab] OR basophil*[tiab] OR eosinophil*[tiab] OR neutrophil*[tiab] OR “natural killer cell*”[tiab] OR “NK cell*”[tiab] OR “T cell*”[tiab] OR “T lymphocyte*”[tiab] OR “T helper cell*”[tiab] OR “infiltrating lymphocyte*”[tiab] OR “T lymphoid cell*”[tiab] OR “T regulatory lymphocyte*”[tiab] OR “T regulatory cell*”[tiab] OR Treg*[tiab] OR “T reg”[tiab] OR “T regs”[tiab] OR monocyte*[tiab] OR macrophage*[tiab] OR TAM[tiab] OR TAMs[tiab] OR BMDM*[tiab] OR “peripheral blood mononuclear cell*”[tiab] OR PBMC*[tiab] OR “antigen presenting cell*”[tiab] OR APC[tiab] OR APCs[tiab] OR “mast cell*”[tiab] OR “glymphatic system*”[tiab] OR “lymphatic system*”[tiab] OR “lymphoid system*”[tiab] OR “lymphatic vessel*”[tiab] OR astrocyte*[tiab] OR microglia[tiab] OR oligodendrocyte*[tiab] OR oligodendroglia[tiab] OR fibroblast*[tiab] OR “mesenchymal stem cell*”[tiab] OR MSC[tiab] OR MSCs[tiab] OR “stromal cell*”[tiab] OR “endothelial cell*”[tiab] OR endothelium[tiab] OR microvasculature[tiab] OR angiogenesis[tiab] OR vasculature*[tiab] OR angiogenesis[tiab] OR neoangiogenesis[tiab] OR “blood vessel*”[tiab] OR neuron*[tiab] OR neurone*[tiab] OR neuronal[tiab] OR “blood brain barrier*”[tiab] OR BBB[tiab] OR “extracellular matrix”[tiab] OR ECM[tiab] OR “immune therap*”[tiab] OR immunotherap*[tiab] OR “immune checkpoint”[tiab] OR pembrolizumab[tiab] OR nivolumab[tiab] OR ipilimumab[tiab] OR atezolizumab[tiab] OR avelumab[tiab] OR durvalumab[tiab] OR cemiplimab[tiab] OR “dendritic cell vaccine*”[tiab] OR “DC vaccine*”[tiab] OR “peptide vaccine*” OR rindopepimut[tiab] OR “CSF 1R” OR “GM CSF” OR bevacizumab[tiab] OR virotherap*[tiab] OR “viral therap*”[tiab]

Concept 2**:** brain metastasis

*Keywords*:

“brain metastas*”[tiab] OR “brain met”[tiab] OR “brain mets”[tiab] OR “cerebral metastas*”[tiab] OR “intracerebral metastas*”[tiab] OR “cerebellar metastas*”[tiab] OR “intracerebellar metastas*”[tiab] OR “intracranial metastas*”[tiab] OR “secondary brain tumour*”[tiab] OR “secondary brain tumor*”[tiab] OR “secondary brain cancer*”[tiab] OR “secondary brain neoplasm*”[tiab] OR “metastatic brain tumour*”[tiab] OR “metastatic brain tumor*”[tiab] OR “metastatic brain cancer*”[tiab] OR “metastatic brain neoplasm*”[tiab] OR “brain metastatic disease*”[tiab]

Construction:

1. “Tumor Microenvironment”[Mesh] OR “Immune System”[Mesh] OR “Hematopoietic System”[Mesh] OR “Neuroglia”[Mesh] OR “Connective Tissue Cells”[Mesh] OR “Endothelial Cells”[Mesh] OR “Neurons”[Mesh] OR “Blood-Brain Barrier”[Mesh] OR “Blood Vessels”[Mesh] OR “Neovascularization, Physiologic”[Mesh] OR “Extracellular Space”[Mesh] OR “Immunotherapy”[Mesh] OR “Antineoplastic Agents, Immunological”[Mesh] OR “Immunotherapy, Adoptive”[Mesh] OR “Antibodies, Monoclonal, Humanized”[Mesh] OR “Cancer Vaccines”[Mesh] OR “Angiogenesis Inhibitors”[Mesh] OR “Oncolytic Virotherapy”[Mesh] OR TME[tiab] OR microenvironment*[tiab] OR “B lymphocyte*”[tiab] OR “B cell*”[tiab] OR “plasma cell*”[tiab] OR “dendritic cell*”[tiab] OR granulocyte*[tiab] OR basophil*[tiab] OR eosinophil*[tiab] OR neutrophil*[tiab] OR “natural killer cell*”[tiab] OR “NK cell*”[tiab] OR “T cell*”[tiab] OR “T lymphocyte*”[tiab] OR “T helper cell*”[tiab] OR “infiltrating lymphocyte*”[tiab] OR “T lymphoid cell*”[tiab] OR “T regulatory lymphocyte*”[tiab] OR “T regulatory cell*”[tiab] OR Treg*[tiab] OR “T reg”[tiab] OR “T regs”[tiab] OR monocyte*[tiab] OR macrophage*[tiab] OR TAM[tiab] OR TAMs[tiab] OR BMDM*[tiab] OR “peripheral blood mononuclear cell*”[tiab] OR PBMC*[tiab] OR “antigen presenting cell*”[tiab] OR APC[tiab] OR APCs[tiab] OR “mast cell*”[tiab] OR “glymphatic system*”[tiab] OR “lymphatic system*”[tiab] OR “lymphoid system*”[tiab] OR “lymphatic vessel*”[tiab] OR astrocyte*[tiab] OR microglia[tiab] OR oligodendrocyte*[tiab] OR oligodendroglia[tiab] OR fibroblast*[tiab] OR “mesenchymal stem cell*”[tiab] OR MSC[tiab] OR MSCs[tiab] OR “stromal cell*”[tiab] OR “endothelial cell*”[tiab] OR endothelium[tiab] OR microvasculature[tiab] OR angiogenesis[tiab] OR vasculature*[tiab] OR angiogenesis[tiab] OR neoangiogenesis[tiab] OR “blood vessel*”[tiab] OR neuron*[tiab] OR neurone*[tiab] OR neuronal[tiab] OR “blood brain barrier*”[tiab] OR BBB[tiab] OR “extracellular matrix”[tiab] OR ECM[tiab] OR “immune therap*”[tiab] OR virotherap*[tiab] OR “immune checkpoint”[tiab] OR pembrolizumab[tiab] OR nivolumab[tiab] OR ipilimumab[tiab] OR atezolizumab[tiab] OR avelumab[tiab] OR durvalumab[tiab] OR cemiplimab[tiab] OR “dendritic cell vaccine*”[tiab] OR “DC vaccine*”[tiab] OR “peptide vaccine*” OR rindopepimut[tiab] OR “CSF 1R” OR “GM CSF” OR bevacizumab[tiab] OR virotherap*[tiab] OR “viral therap*”[tiab]
2. “brain metastas*”[tiab] OR “brain met”[tiab] OR “brain mets”[tiab] OR “cerebral metastas*”[tiab] OR “intracerebral metastas*”[tiab] OR “cerebellar metastas*”[tiab] OR “intracerebellar metastas*”[tiab] OR “intracranial metastas*”[tiab] OR “secondary brain tumour*”[tiab] OR “secondary brain tumor*”[tiab] OR “secondary brain cancer*”[tiab] OR “secondary brain neoplasm*”[tiab] OR “metastatic brain tumour*”[tiab] OR “metastatic brain tumor*”[tiab] OR “metastatic brain cancer*”[tiab] OR “metastatic brain neoplasm*”[tiab] OR “brain metastatic disease*”[tiab]
3. #1 AND #2

In one string:

(“Tumor Microenvironment”[Mesh] OR “Immune System”[Mesh] OR “Hematopoietic System”[Mesh] OR “Neuroglia”[Mesh] OR “Connective Tissue Cells”[Mesh] OR “Endothelial Cells”[Mesh] OR “Neurons”[Mesh] OR “Blood-Brain Barrier”[Mesh] OR “Blood Vessels”[Mesh] OR “Neovascularization, Physiologic”[Mesh] OR “Extracellular Space”[Mesh] OR “Immunotherapy”[Mesh] OR “Antineoplastic Agents, Immunological”[Mesh] OR “Immunotherapy, Adoptive”[Mesh] OR “Antibodies, Monoclonal, Humanized”[Mesh] OR “Cancer Vaccines”[Mesh] OR “Angiogenesis Inhibitors”[Mesh] OR “Oncolytic Virotherapy”[Mesh] OR TME[tiab] OR microenvironment*[tiab] OR “B lymphocyte*”[tiab] OR “B cell*”[tiab] OR “plasma cell*”[tiab] OR “dendritic cell*”[tiab] OR granulocyte*[tiab] OR basophil*[tiab] OR eosinophil*[tiab] OR neutrophil*[tiab] OR “natural killer cell*”[tiab] OR “NK cell*”[tiab] OR “T cell*”[tiab] OR “T lymphocyte*”[tiab] OR “T helper cell*”[tiab] OR “infiltrating lymphocyte*”[tiab] OR “T lymphoid cell*”[tiab] OR “T regulatory lymphocyte*”[tiab] OR “T regulatory cell*”[tiab] OR Treg*[tiab] OR “T reg”[tiab] OR “T regs”[tiab] OR monocyte*[tiab] OR macrophage*[tiab] OR TAM[tiab] OR TAMs[tiab] OR BMDM*[tiab] OR “peripheral blood mononuclear cell*”[tiab] OR PBMC*[tiab] OR “antigen presenting cell*”[tiab] OR APC[tiab] OR APCs[tiab] OR “mast cell*”[tiab] OR “glymphatic system*”[tiab] OR “lymphatic system*”[tiab] OR “lymphoid system*”[tiab] OR “lymphatic vessel*”[tiab] OR astrocyte*[tiab] OR microglia[tiab] OR oligodendrocyte*[tiab] OR oligodendroglia[tiab] OR fibroblast*[tiab] OR “mesenchymal stem cell*”[tiab] OR MSC[tiab] OR MSCs[tiab] OR “stromal cell*”[tiab] OR “endothelial cell*”[tiab] OR endothelium[tiab] OR microvasculature[tiab] OR angiogenesis[tiab] OR vasculature*[tiab] OR angiogenesis[tiab] OR neoangiogenesis[tiab] OR “blood vessel*”[tiab] OR neuron*[tiab] OR neurone*[tiab] OR neuronal[tiab] OR “blood brain barrier*”[tiab] OR BBB[tiab] OR “extracellular matrix”[tiab] OR ECM[tiab] OR “immune therap*”[tiab] OR virotherap*[tiab] OR “immune checkpoint”[tiab] OR pembrolizumab[tiab] OR nivolumab[tiab] OR ipilimumab[tiab] OR atezolizumab[tiab] OR avelumab[tiab] OR durvalumab[tiab] OR cemiplimab[tiab] OR “dendritic cell vaccine*”[tiab] OR “DC vaccine*”[tiab] OR “peptide vaccine*” OR rindopepimut[tiab] OR “CSF 1R” OR “GM CSF” OR bevacizumab[tiab] OR virotherap*[tiab] OR “viral therap*”[tiab]) AND (“brain metastas*”[tiab] OR “brain met”[tiab] OR “brain mets”[tiab] OR “cerebral metastas*”[tiab] OR “intracerebral metastas*”[tiab] OR “cerebellar metastas*”[tiab] OR “intracerebellar metastas*”[tiab] OR “intracranial metastas*”[tiab] OR “secondary brain tumour*”[tiab] OR “secondary brain tumor*”[tiab] OR “secondary brain cancer*”[tiab] OR “secondary brain neoplasm*”[tiab] OR “metastatic brain tumour*”[tiab] OR “metastatic brain tumor*”[tiab] OR “metastatic brain cancer*”[tiab] OR “metastatic brain neoplasm*”[tiab] OR “brain metastatic disease*”[tiab]) NOT (Review[Publication Type] OR Systematic Review[Publication Type] OR Meta-Analysis[Publication Type])

Search results:

1^st^ search: 22^nd^ June 2022

🡪 search results: 3083

2^nd^ search: 22^nd^ March 2024

🡪 search results: 742; 3083 + 742 = 3825

3^rd^ search: 25^th^ September 2024

🡪 search results: 248; 3083 + 742 + 248 = 4073

4^th^ search: 11^th^ September 2025

🡪 search results: 512; 3083 + 742 + 248 + 512 = 4585
